# Xbra and Smad-1 cooperate to activate the transcription of neural repressor *ventx1*.*1* in *Xenopus* embryos

**DOI:** 10.1038/s41598-018-29740-9

**Published:** 2018-07-30

**Authors:** Shiv Kumar, Zobia Umair, Jaeho Yoon, Unjoo Lee, Sung Chan Kim, Jae-Bong Park, Jae-Yong Lee, Jaebong Kim

**Affiliations:** 1 Department of Biochemistry, Institute of Cell Differentiation and Aging, College of Medicine, Hallym University, Chuncheon, Gangwon-Do 24252 Republic of Korea; 2 Department of Electrical Engineering, Hallym University, Chuncheon, Gangwon-Do 24252 Republic of Korea

## Abstract

Crosstalk of signaling pathways play crucial roles in cell proliferation, cell differentiation, and cell fate determination for development. In the case of *ventx1*.*1* in *Xenopus* embryos, both BMP-4/Smad-1 and FGF/Xbra signaling induce the expression of neural repressor *ventx1*.*1*. However, the details of how these two pathways interact and lead to neural inhibition by *ventx1*.*1* remain largely unknown. In the present study, Xbra directly bound to the *ventx1*.*1* promoter region and inhibited neurogenesis in a Ventx1.1-dependent manner. Furthermore, Smad-1 and Xbra physically interacted and regulated *ventx1*.*1* transcription in a synergistic fashion. Xbra and Smad-1 interaction cooperatively enhanced the binding of an interacting partner within the *ventx1*.*1* promoter and maximum cooperation was achieved in presence of intact DNA binding sites for both Smad-1 and Xbra. Collectively, BMP-4/Smad-1 and FGF/Xbra signal crosstalk cooperate to activate the transcription of neural repressor *ventx1*.*1* in *Xenopus* embryos. This suggests that the crosstalk between BMP-4 and FGF signaling negatively regulates early neurogenesis by synergistic activation of *ventx1*.*1* in *Xenopus* embryos.

## Introduction

Bone morphogenetic protein-4 (BMP-4) signaling is actively involved in germ-layer specification, axis formation and cell differentiation in *Xenopus*^1,2^, and blocking of BMP-4 signaling is achieved via organizer-secreted BMP antagonists, including Chordin, Noggin, Follistatin, and Cerberus results in neural induction^3^. Previous study demonstrated that BMP-4/Smad-1 directly induces the *ventx1*.*1* (*PV*.*1*, *Xvent-1b*) expression in *Xenopus* embryos and leads to ventral mesoderm and ectoderm formation^4^. *Ventx1*.*1* is one of the homeobox transcriptional factors of Xvent family^5^ and locates to Ventx synteny in *Xenopus*^6^. Ventx synteny is evolutionary conserved in human, dog, chicken and *Xenopus* genomes, but it is absent in genomes of rat and mouse^6^. Studies have reported that *ventx1*.*1* is an endogenous neural inhibitor in *Xenopus*, inhibiting the expression of organizer and early neural genes, including *Chordin*, *Noggin*, *FoxD5a/b*, *gsc* and *Zic3* via its C-terminal^5,7,8^. Additionally, ectopic expression of *ventx1*.*1* not only inhibits neural genes expression but also induces the expression of ventral genes, including wnt8 and xhox3 and results in the headless phenotype along with neural inhibition in *Xenopus*^5,7,9^. Previous studies have demonstrated that Sox2/3 and geminin regulates the proliferation and differentiation of neural progenitor cells^10,11^. The expressions for *Sox2/3* and *geminin* are positively regulated by Wnt/b-catenin signaling while being negatively regulated by BMP-4 signaling in *Xenopus* embryos. Ectopic expression of ventx1/2 inhibits endogenous expression of the early neural genes, namely *Sox2/3* and *geminin* in *Xenopus* embryos, causing the neural inhibition. Furthermore, knockdown of *ventx1/2* triggers the expression of *Sox2/3* and *geminin* in *Xenopus* embryos, resulting in neural induction.

FGF/Xbra signaling actively participates in cell fate determination and anterior-posterior (A-P) patterning of neural tissue^12^. FGF leads mesoderm formation through the activation of an autocatalytic loop (FGF/Ras/Xbra/AP-1) in *Xenopus*^12,13^. Xbra is a member of the T-box gene family and a downstream transcriptional activator of FGF signaling. Ectopic expression of Xbra triggers mesoderm formation and inhibits neurogenesis in *Xenopus* embryos. Our previous study demonstrated that the ectopic expression of dominant negative Xbra (DN-Xbra) inhibits *ventx1*.*1* expression and triggers expression of *chordin* and neural marker genes, including *Zic3*, *Otx2*, and *N-CAM* in animal cap explants^14^. This study suggested that FGF/Xbra may negatively regulate neurogenesis in a Ventx1.1-dependent manner in *Xenopus* embryos. However, the detailed molecular mechanism of the Xbra-mediated transcriptional activation of *ventx1*.*1* and its involvement in neural inhibition is not completely understood.

Recent studies documented that FGF signaling leads to both activation and inhibition in neural induction in *Xenopus* embryos^14,15^. FGF/MAPK enhanced neural induction by inhibiting BMP-4/Smad-1 signaling in vertebrates^15^. However, the FGF-mediated BMP-4/Smad-1 inhibition alone was not sufficient to trigger neurogenesis in *Xenopus* embryos^3^. Our previous study has demonstrated that the bFGF-treatment, where *Xbra* expresses (with it being a kind of a mesoderm tissue), induces the *ventx1*.*1* expression in animal cap explants, resulting in neural inhibition^14^. The knockdown of *ventx1*.*1* by *ventx1*.*1* morpholino causes the neurogenesis by inducing the expression of early and late neural genes, including *Zic3*, *NCAM*, *Otx2*, *Rx1* and *HoxB9* in bFGF-treated ectodermal explants of *Xenopus* embryos, suggesting that the BMP-4/Smad-1 and FGF/Xbra may participate in a signaling crosstalk to inhibit neurogenesis in a Ventx1.1-dependent manner^4,14^. Recent studies have indicated that T-box transcriptional factors bind to the consensus sequence (A/G)(A/T)(A/T)NTN(A/G)CAC(C/T)T and positively regulate expression of genes, including e*FGF*, *Bix4* and *Bix1*^16–18^. Another study reported that phosphorylated C-terminal of Smad-1 physically interacts with the N-terminal containing HLL(S/N)AV(E/Q) motif of Xbra in *Xenopus*^19^. Additionally, the study suggested that Smad-1-Xbra interaction may induce the expression of ventral genes. However, the detailed molecular mechanism that allows BMP-4/Smad-1 and FGF/Xbra-mediated signaling crosstalk to inhibit neurogenesis in a Ventx1.1-dependent manner is largely unknown.

In the present study, we demonstrated that Xbra directly binds to Xbra response elements, ATCACACTT (XbRE, within −70 bp~−62 bp) upstream of the putative transcription initiation site (TSS) of *ventx1*.*1* and positively induces *ventx1*.*1* transcription. The results showed that Xbra and Smad-1 synergistically cooperate to regulate *ventx1*.*1* transcription during *Xenopus* development. ChIP-PCR assays indicated that Smad-1 and Xbra directly bind to their respective consensus sequences within the proximal region of the endogenous *ventx1*.*1* promoter. Additionally, Xbra and Smad-1 enhanced the binding of their respective interacting partners to the promoter region. We further observed that the Xbra and Smad-1 interaction displays target specificity in the Xvent family as Xbra and Smad-1 did not synergistically induce *Xvent2* expression. Taken together, it is concluded that BMP-4/Smad-1 and FGF/Xbra signaling demonstrate a crosstalk for inhibition of neurogenesis in a Ventx1.1-dependent manner in *Xenopus* embryos. These results suggest that FGF/Xbra and BMP-4/Smad-1 are positively involved in the transcriptional activation of *ventx1*.*1* and synergistically cooperate to inhibit neurogenesis in the ventral and lateral mesoderm in *Xenopus* embryos.

## Results

### Ectopic expression of Xbra inhibits neurogenesis in a Ventx1.1-dependent manner in animal cap explants of *Xenopus*

**Figure 1:**
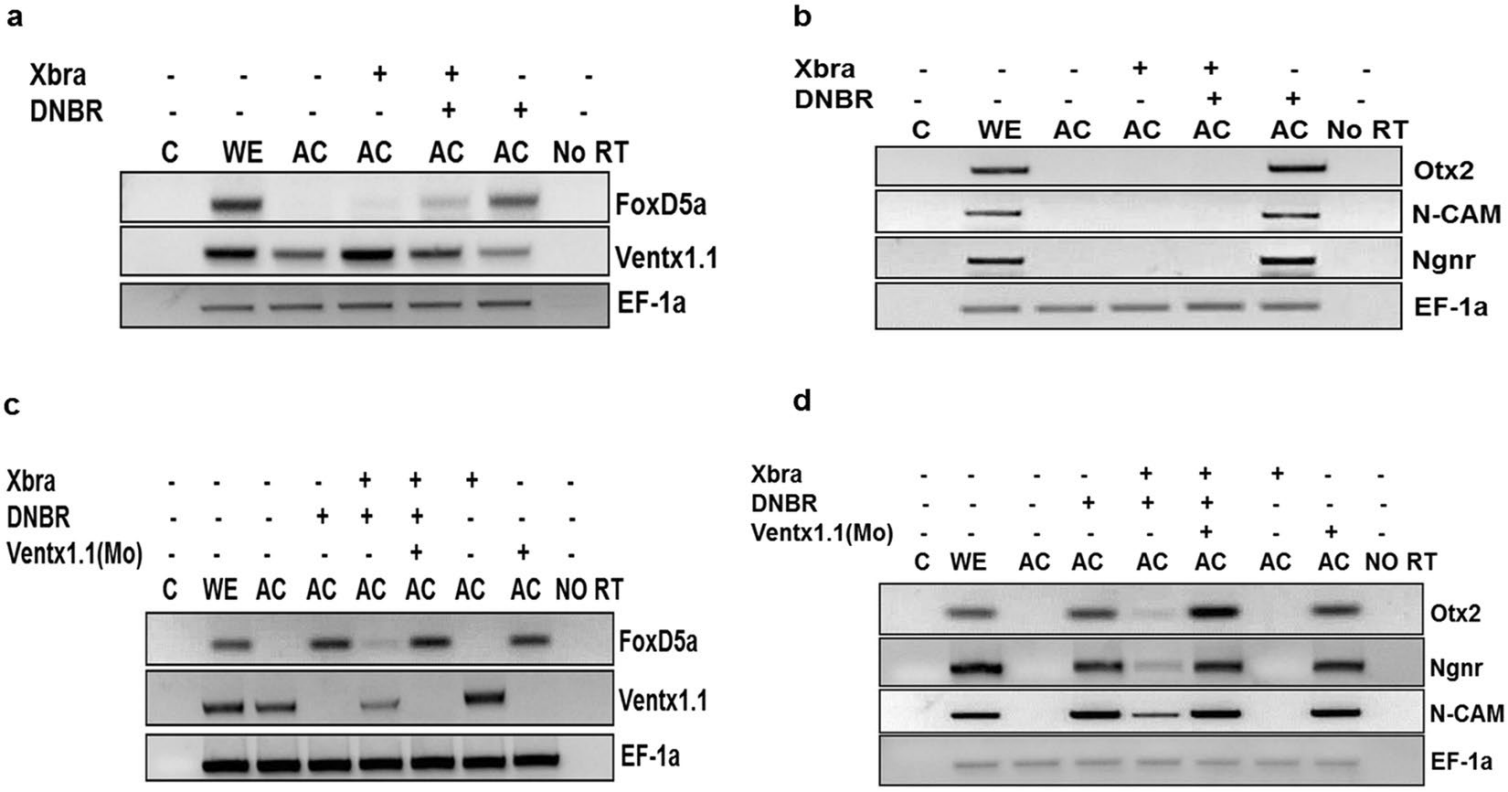
Ectopic expression of Xbra inhibits neurogenesis in a Ventx1.1-dependent manner in animal cap explants of *Xenopus*. *Ventx1*.*1* MOs (1 ng) were injected either with or without DNBR and Xbra mRNA at the 1-cell stage and dissected the animal cap at the stage 8. The dissected animal caps were harvested until stages 11 and 24 in L-15 culture medium. The relative gene expressions were analyzed by RT-PCR. (**a**,**b**) Xbra increases *ventx1*.*1* expression and reduces the expression of early and late neural genes, including that of *FoxD5a*, *N-CAM*, *Ngnr*, and *Otx2* while DNBR reduces *ventx1*.*1* expression and induces the expression of neural genes, *FoxD5a*, *N-CAM*, *Ngnr*, and *Otx2*. (**c**,**d**) *ventx1*.*1* MOs reduces *ventx1*.*1* expression and increases the expression of neural genes, including for *FoxD5a*, *N-CAM*, *Ngnr*, and *Otx2*. C: PCR reaction without adding cDNA while No RT means PCR reaction without added reverse transcriptase.

Previous studies demonstrated that both BMP-4/Smad-1 and FGF/Xbra signaling induce *ventx1*.*1* expression which in turn negatively regulates neurogenesis in *Xenopus* embryos^4,14^. Studies have documented that Xbra is also induced by BMP-4 in *Xenopus* and human embryonic stem cells, leading to mesoderm differentiation^20^. Thus, we examined whether Xbra induces *ventx1*.*1* expression under a BMP-4-inhibited condition and causes neural inhibition. Dominant-negative BMP-4 receptor (DNBR) mRNA was injected for BMP-4 inhibition. RT-PCR results showed Xbra induced *ventx1*.*1* expression under the BMP-4-inhibited condition and suppressed early and late neural genes including *FoxD5a*, *Ngnr*, *N-CAM* and *Otx2* in animal cap explants (Fig. 1a,b). The results suggested that Xbra positively regulates *ventx1*.*1* expression in a BMP-inhibited condition and inhibits neurogenesis in animal cap explants of *Xenopus* embryos. However, it was still questionable whether Xbra-mediated neural inhibition is truly dependent on Ventx1.1. Thus, we performed a knockdown of *ventx1*.*1* by *ventx1*.*1* morpholino (MOs) and found that the knockdown of *ventx1*.*1* strongly augments and recovers the expression of both early and late neural genes including *FoxD5a*, *N-CAM*, *Ngnr*, and *Otx2*, which were reduced by overexpression of Xbra (Fig. 1c,d). Moreover, *ventx1*.*1* MOs specifically reduced *ventx1*.*1* expression in both the absence and presence of Xbra. These results showed that Xbra negatively regulates neurogenesis in a Ventx1.1-dependent manner. Taken together, these results indicated that Xbra induces *ventx1*.*1* expression in ventral and lateral mesodermal zones, leading to inhibition of expression of early neural genes and causing neural inhibition in part of the mesodermal region. In addition, the 5′-flanking region of *ventx1*.*1* likely contains an Xbra response element (XbRE) which directly regulates *ventx1*.*1* transcription. Moreover, *ventx1*.*1* establishes signaling crosstalk between BMP-4/Smad-1 and FGF/Xbra in order to inhibit neurogenesis in *Xenopus* embryos.

### Xbra response element (XbRE) is identified within the proximal region of the *ventx1*.*1* promoter

**Figure 2:**
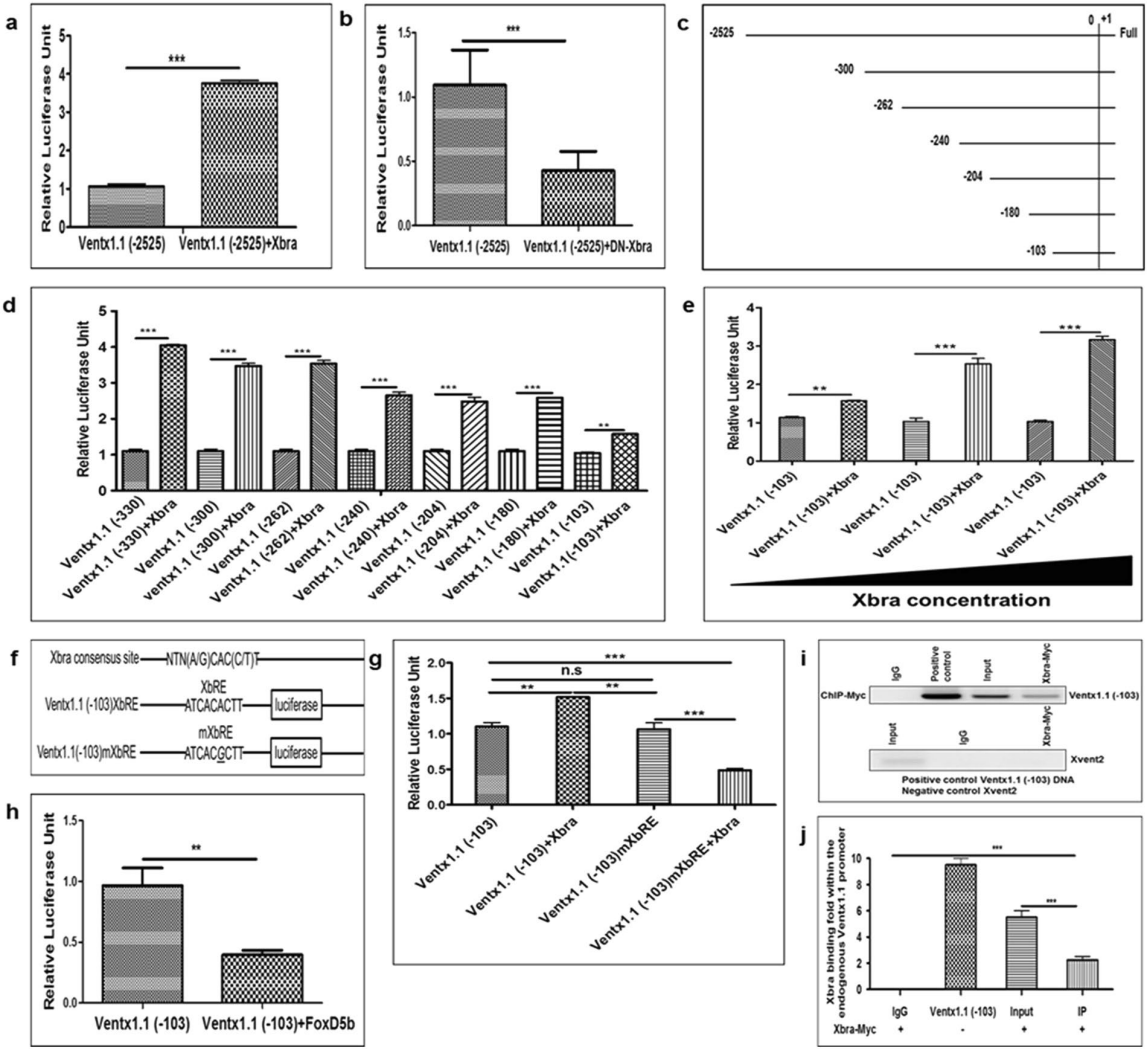
Identification of Xbra response elements (XbRE) within the *ventx1*.*1* promoter region. Different serially-deleted *ventx1*.*1* promoter constructs were co-injected either with or without DN-Xbra or Xbra at the 1-cell stage and grown until stage 11 in 30% MMR to measure the relative promoter activity at the stage 11. (**a**) *ventx1*.*1* (−2525) promoter injected either with or without DN-Xbra. (**b**) *ventx1*.*1* (−2525) promoter injected either with or without Xbra. (**c**,**d**) Serially-deleted promoter constructs of *ventx1*.*1* injected either with or without Xbra. (**e**) *ventx1*.*1* (−103) promoter construct injected either with or without Xbra in a dose-dependent manner. (**f**) Putative Xbra binding consensus was mutated by site-directed mutagenesis within *ventx1*.*1* (−103) promoter constructs. (**g**) XbRE-mutated *ventx1*.*1* (−103) mXbRE and *ventx1*.*1* (−103) promoter constructs injected either with or without Xbra. (**h**) *ventx1*.*1* (−103) promoter construct injected either with or without FoxD5b. (**i**,**j**) A ChIP-PCR assay was performed with anti-Myc antibody during gastrula. All ChIP bindings were measured by PCR using specific primers. *ventx1*.*1* (−103) promoter DNA was used as a positive control while Xvent2 coding region primers were used as a negative control for all the ChIP-PCR experiments. All the relative promoter activity data are shown as mean ± SE. The ChIP-PCR band intensities were quantified using a densitometer.

The results (Fig. 1) suggested that the *ventx1*.*1* promoter region may contain a putative cis-acting XbRE which is required for triggering the *ventx1*.*1* transcription. To evaluate the presence of XbRE within the *ventx1*.*1* promoter region, we injected a *ventx1*.*1* (−2525) promoter construct either with or without DN-Xbra, and Xbra, separately. These results showed that DN-Xbra decreased the relative promoter activity of *ventx1*.*1* (−2525) up to 2.5-fold, while Xbra increased the relative promoter activity of *ventx1*.*1* (−2525) up to 3.5-fold compared to that of *ventx1*.*1* (−2525) construct alone (Fig. 2a,b). The results showed that Xbra positively regulates *ventx1*.*1* transcription and the *ventx1*.*1* promoter may contain a putative cis-acting XbRE. Therefore, we generated different serially-deleted *ventx1*.*1* promoter constructs (Fig. 2c), which were co-injected either with or without Xbra, separately. The results showed that Xbra increases the relative promoter activity of serially-deleted *ventx1*.*1* promoter constructs up to 1.5-4.0-fold compared to those of *ventx1*.*1* promoter without Xbra (Fig. 2d). Moreover, Xbra increased the relative promoter activity of *ventx1*.*1* (−103) in a dose-dependent manner (Fig. 2e). These results strongly provided evidence that the *ventx1*.*1* (−103) promoter construct contained a cis-acting XbRE which induces *ventx1*.*1* transcription in *Xenopus* embryos.

Previous studies have addressed the putative binding consensus sequence (A/G)(A/T)(A/T)NTN(A/G)CAC(C/T)T of T-box transcription factors^21^ within the promoter region of targeted genes, including e*FGF*, *Bix4*, and *Bix1*^16–18^. Thus, we mapped the *ventx1*.*1* (−103) promoter nucleotide sequence and found a putative XbRE, (ATCACACTT, within −70 bp~−62 bp), upstream of putative TSS of *ventx1*.*1*. We mutated one nucleotide (changed ATCACACTT to ATCAC*G*CTT) within the putative XbRE in *ventx1*.*1* (−103) promoter construct and generated XbRE-mutated *ventx1*.*1* (−103) mXbRE (Fig. 2f). We then tested the reporter gene activities of *ventx1*.*1* (−103) and *ventx1*.*1* (−103) mXbRE promoter constructs either with or without Xbra. The results showed that Xbra-mediated transcriptional activation of *ventx1*.*1* (−103) was abolished in *ventx1*.*1* (−103) mXbRE (Fig. 2g). Xbra significantly reduced and did not increase the relative promoter activity of XbRE-mutated *ventx1*.*1* (−103) promoter construct. We also tested whether the *ventx1*.*1* (−103) promoter construct contains the negative response element of early neural protein FoxD5b. Result showed that FoxD5b significantly reduces the relative promoter activity of *ventx1*.*1* (−103) (Fig. 2h).

The results firmly indicated that *ventx1*.*1* (−103) promoter construct contains a cis-acting XbRE in the proximal region (−70 bp~−62 bp) of the *ventx1*.*1* promoter. Previous studies have documented that Xbra directly binds within the promoter regions of Xvent family of genes for *Xenopus* and human^22–24^. A genome-wide ChIP-Seq study of T-box transcription factors in *X*. *tropicalis* has shown that Ventx family is one of the direct targets of T-box family transcription factors. We then performed ChIP-PCR assay and found that Xbra directly bound within the proximal region of the endogenous *ventx1*.*1* promoter (Fig. 2i,j). These findings collectively concluded that *ventx1*.*1* is one of the direct targets of Xbra and the direct binding of Xbra within the proximal promoter region increases transcription activity of *ventx1*.*1*.

### Xbra and Smad-1 synergistically regulate *ventx1*.*1* transcription

**Figure 3:**
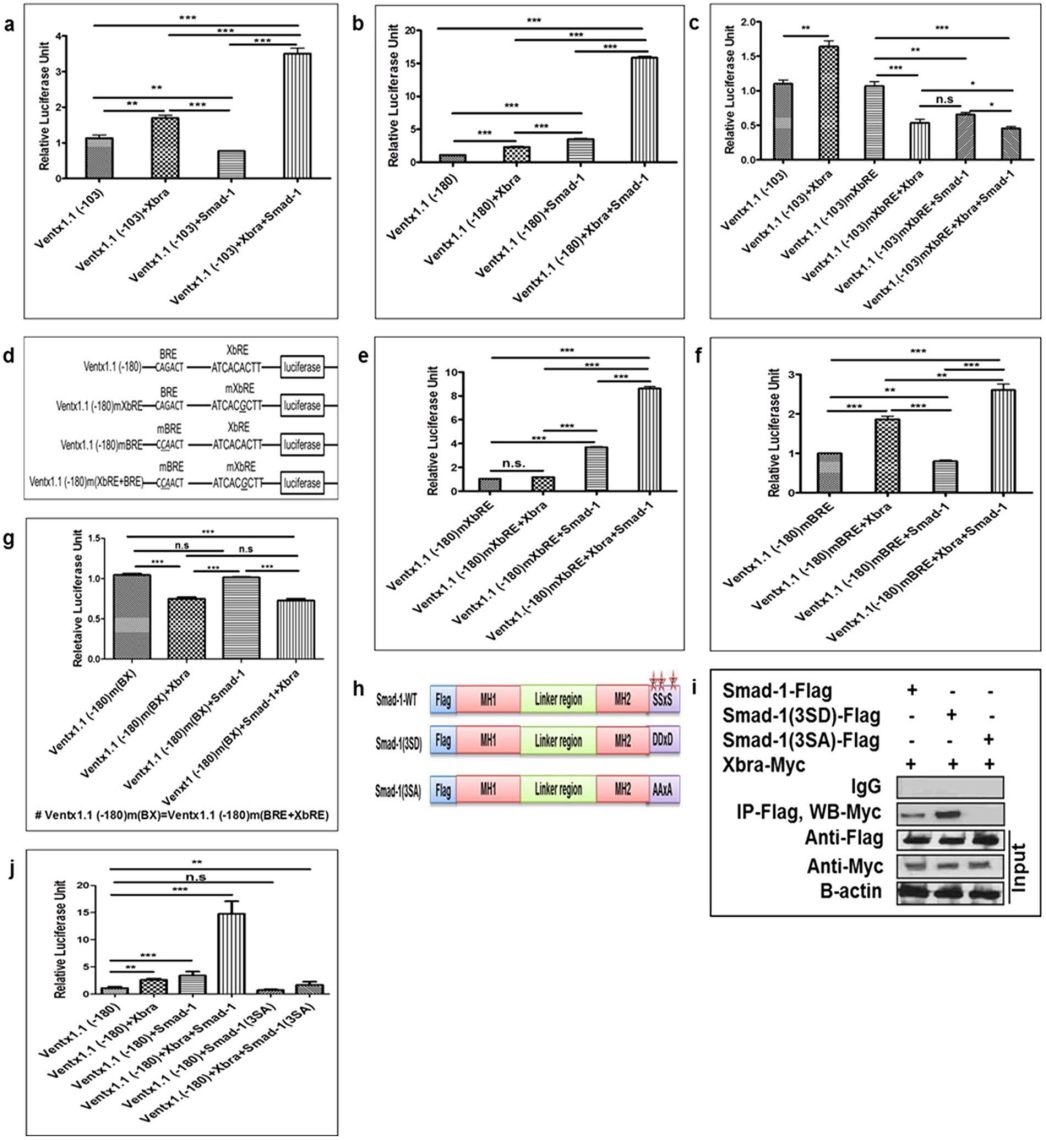
Xbra and Smad-1 synergistically regulate *ventx1*.*1* transcription. Different *ventx1*.*1* promoter constructs were co-injected either with or without Smad-1 and with Xbra, either in combination or separately, at the 1-cell stage and grown until stage 11 to measure the relative promoter activity. (**a**,**b**) *ventx1*.*1* (−103 and −180) injected with Xbra and Smad-1, in combination or separately. (**c**) XbRE-mutated *ventx1*.*1* (−103) mXbRE and *ventx1*.*1* (−103) injected with Xbra and Smad-1, in combination or separately, in separate groups. (**d**) XbRE and BRE were mutated by site-directed mutagenesis in different *ventx1*.*1* (−180) promoter constructs. (**e**) XbRE-mutated *ventx1*.*1* (−180) mXbRE injected either with or without Xbra and Smad-1, in combination or separately. (**f**) BRE-mutated *ventx1*.*1* (−180) mBRE injected either with or without Xbra and Smad-1, in combination or separately. (**g**) The doubly mutated *ventx1*.*1* (−180) m(BRE + XbRE) injected either with or without Xbra and Smad-1, in combination or separately. (**h**,**i**) Flag-Smad-1, Flag-Smad-1 (3SD), and Flag-Smad-1 (3SA) constructs were co-injected with Myc-Xbra and immunoprecipitation was performed with an anti-Flag antibody. (**j**) *ventx1*.*1* (−180) injected either with or without Myc-Xbra, Flag-Smad-1, and Flag-Smad-1 (3SA) constructs in different groups, in combination or separately. All relative promoter activity data are shown as mean ± SE.

*Lee H*. *S*. *et al*.^4^ demonstrated that Smad-1 directly binds to cis-acting BMP-4 response elements (BRE; CAGACT, −180 bp to −162 bp) within the *ventx1*.*1* promoter^4^. Our findings showed that both Xbra and Smad-1 positively regulate *ventx1*.*1* expression^4,14^ and two cis-acting elements (BRE; −180 bp to −162 bp and XbRE; −70 bp to −62 bp) exist within the proximal promoter region of the *ventx1*.*1*. Thus, we further examined whether Xbra and Smad-1 synergistically cooperate to activate *ventx1*.*1* transcription in *Xenopus* embryos. We co-injected the *ventx1*.*1* (−103 and −180) promoter constructs either with or without Xbra and Smad-1, in a combination or separately. The *ventx1*.*1* (−103) construct contains only the XbRE, while the *ventx1*.*1* (−180) construct contains both the cis-acting elements BRE and XbRE. The results showed that co-injection of Smad-1 and Xbra mRNA increased the relative promoter activities of *ventx1*.*1* (−103 and −180) up to 3.5-fold and 16-fold compared to those of *ventx1*.*1* (−103 and −180) alone, respectively (Fig. 3a,b). Xbra or Smad-1 alone did not lead to the maximal increase (16-fold) in the relative promoter activities neither with *ventx1*.*1* (−103) nor with *ventx1*.*1* (−180) promoter constructs. Collectively, the results strongly indicated that Smad-1 and Xbra synergistically cooperate to activate *ventx1*.*1* transcription. To examine the DNA sequence required for Xbra and Smad-1-mediated synergistic regulation of *ventx1*.*1* transcription, we injected the *ventx1*.*1* (−103) and XbRE-mutated *ventx1*.*1* (−103) promoter (*ventx1*.*1* (−103) mXbRE) constructs with both Xbra and Smad-1 in combination or separately. With the *ventx1*.*1* (−103) mXbRE construct, the Xbra and Smad-1-mediated stimulation of the *ventx1*.*1* (−103) promoter construct was completely abolished (Fig. 3c, bar 4–6 vs. bar 2), indicating that the cis-acting element of XbRE is essential for Xbra-mediated activation as well as Xbra and Smad-1-mediated synergistic activation of *ventx1*.*1* transcription.

The concomitant overexpression of Xbra and Smad-1 increased the relative promoter activity of *ventx1*.*1* (−103 and −180) for up to 3.5-fold and 16-fold as compared with those of *ventx1*.*1* (−103 and −180) alone, respectively (Fig. 3a,b, bar 4). We then examined whether both of the cis-acting elements (BRE and XbRE) were equally important in the synergistic activation of *ventx1*.*1* transcription. We mutated BRE and XbRE within the *ventx1*.*1* (−180) promoter construct and generated three different mutated promoter constructs of *ventx1*.*1* (−180); these were the XbRE-mutated *ventx1*.*1* (−180) mXbRE, the BRE-mutated *ventx1*.*1* (−180) mBRE and the doubly mutated *ventx1*.*1* (−180) m(BRE + XbRE) (Fig. 3d). The *ventx1*.*1* (−180) mXbRE promoter construct was co-injected either with or without Smad-1and Xbra in combination or separately. As we expected, the results showed that Xbra-mediated stimulation was completely abolished (Fig. 3e, 2nd bar) while Smad-1-mediated stimulation was sustained with a 3.5-fold induction in presence of Smad-1 (Fig. 3e, 3rd bar). Interestingly, the XbRE-mutated *ventx1*.*1* (−180) mXbRE promoter construct still contained synergistic activation with an 8.5-fold induction when Xbra was co-injected with Smad-1 (Fig. 3e, 4th bar). It was noted that the Xbra and Smad-1-mediated relative promoter activity of *ventx1*.*1* (−180) mXbRE was decreased 8.5-fold compared to a 16-fold increased activity with the *ventx1*.*1* (−180) promoter construct (Fig. 3b, 4th bar). The results indicated that the XbRE contributes to achieve a maximal fold increase (16-fold) in the synergistic activation of *ventx1*.*1* transcription with Smad-1 and Xbra. We next examined the *ventx1*.*1* (−180) mBRE either with or without Smad-1 and Xbra in combination or separately. The results showed that the Smad-1-mediated stimulation was completely abolished (Fig. 3f, 3rd bar), but the Xbra-mediated stimulation was sustained with a 1.5-fold increase in presence of Xbra (Fig. 3f, 2nd bar). The *ventx1*.*1* (−180) mBRE promoter construct still displayed a certain synergistic activation with a 2.5-fold induction when Smad-1 was co-injected with Xbra (Fig. 3f, 4th bar). Additionally, the relative promoter activity of *ventx1*.*1* (−180) mBRE was dramatically decreased to 2.5-fold compared to the 16-fold increase which was achieved by the Smad-1 and Xbra with the *ventx1*.*1* (−180) promoter construct (Fig. 3b, 4th bar). These results collectively provide evidence that BMP-4/Smad-1 and BRE play a more crucial role in the synergistic regulation of *ventx1*.*1* transcription activation than with FGF/Xbra and XbRE. We further examined the doubly mutated *ventx1*.*1* (−180) m(BRE + XbRE) promoter construct with Smad-1 and Xbra in combination or separately. The results showed that the Xbra and Smad-1-mediated stimulation of the promoter activity of the *ventx1*.*1* (−180) promoter construct was completely abolished in the doubly mutated *ventx1*.*1* (−180)m(BRE + XbRE) construct. Both the synergy as well as stimulation were completely abolished in the doubly mutated *ventx1*.*1* (−180) m(BRE + XbRE) construct (Fig. 3g). These results collectively indicated that both the consensus cis-acting BRE and XbRE are required for maximal activation of *ventx1*.*1* transcription.

Studies have reported that the N-terminal of Xbra physically interacts with phosphorylated C-terminal MH2 domain of Smad-1 for both human and *Xenopus* embryos^19,22^. Thus, we examined the physical interaction of Xbra and Smad-1. We co-injected Xbra with different Smad-1 constructs: Smad-1 (wild-type; wt), the C-terminal phospho-mimic Smad-1 (3SD) (constitutively active form), and the phospho-dead Smad-1 (3SA) (inactive form) (Fig. 3h). The results showed that Xbra physically interacts with Smad-1 and Smad-1 (3SD), while the interaction of Xbra with Smad-1 (3SA) was not detected (Fig. 3i). Moreover, Xbra more strongly bound with Smad-1 (3SD) than Smad-1, suggesting that the C-terminal phosphorylation of Smad-1 plays a critical role in the Xbra-Smad-1 interaction. We then inquired on the effect of Smad-1 and Xbra interaction in synergistic regulation of *ventx1*.*1* transcription. When Smad-1 (wt) was co-injected with Xbra, the relative promoter activity of the *ventx1*.*1* (−180) construct increased up to 15-fold compared to that of *ventx1*.*1* (−180) alone. On the other hand, Smad-1 (3SA) and Xbra did not show synergistic activation of *ventx1*.*1* (−180) (Fig. 3j). These results supported the findings of the previous studies, which showed that the C-terminal phosphorylation of Smad-1 is required for the Smad-1-Xbra interaction and it stimulates the expression of the ventral genes^19,25^. Moreover, the results indicated that the Xbra-Smad-1 interaction is required for the establishment of BMP-4/Smad-1 and FGF/Xbra-mediated signaling crosstalk which synergistically regulates the transcription of a specific target gene, namely *ventx1*.*1* in early *Xenopus* embryos.

### Xbra enhances Smad-1 binding affinity on its cis-acting element (BRE) within the *ventx1*.*1* promoter

**Figure 4:**
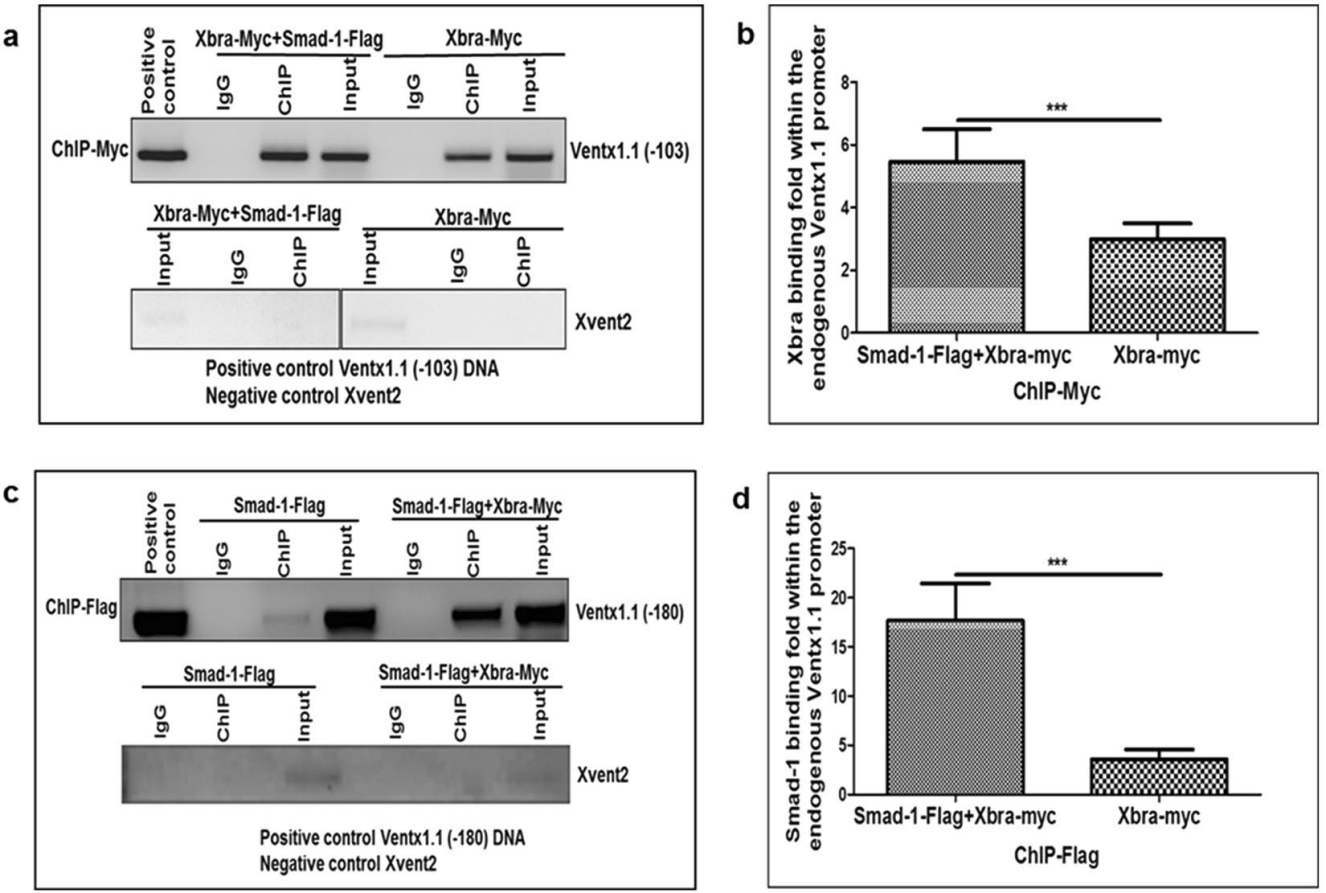
Xbra enhances Smad-1 binding affinity on its cis-acting element (BRE) within the *ventx1*.*1* promoter. To perform ChIP-PCR assays, mRNA was co-injected either with or without Smad-1 at the 1-cell stage and harvested the embryos until stage 11 in 30% MMR. (**a**,**b**) The ChIP-PCR assay was performed with the anti-Myc antibody. (**c**,**d**) The ChIP-PCR assay performed with the anti-Flag antibody at the stage 11. All bindings were measured by PCR method using specific primers. *ventx1*.*1* (−103 and −180) promoter DNA served as positive control. The ChIP-PCR band intensity was quantified using a densitometer.

Smad-1 and Xbra synergistically up-regulated the relative promoter activities of *ventx1*.*1* promoter construct. Therefore, we examined whether Smad-1 and Xbra stimulated the DNA binding of their interacting partner within the proximal region of the endogenous *ventx1*.*1* promoter. We co-injected Myc-Xbra either with or without Smad-1 to perform ChIP-PCR assays with an anti-Myc antibody. The results showed that ectopic expression of Smad-1 increased about 2 fold the Xbra binding intensity within the proximal region of the endogenous *ventx1*.*1* promoter (Fig. 4a,b). We further asked whether Xbra stimulated Smad-1 binding within the proximal region of the endogenous *ventx1*.*1* promoter. We co-injected Flag-Smad-1 either with or without Xbra and performed a ChIP-PCR assay with an anti-Flag antibody. The results showed that Xbra strongly enhances the Smad-1 binding affinity within the proximal region of the endogenous *ventx1*.*1* promoter (Fig. 4c,d). These results collectively indicated that both Xbra and Smad-1 directly bind within the proximal region of the endogenous *ventx1*.*1* promoter. Xbra positively regulates *ventx1*.*1* transcription activation as well as strongly stimulates the Smad-1 binding within the proximal region of the endogenous *ventx1*.*1* promoter.

## Discussion

In this study, we tried to answer two basic questions. The first concerned the presence and the details of the crosstalk between two main signaling pathways, BMP/Smad1 and FGF/Xbra, in the regulation of *ventx1*.*1* expression. The second one was the role and significance of *ventx1*.*1* expression in neural inhibition. In this discussion, we try to put forward the significance of *ventx1*.*1* expression regulated by BMP-4/Smad-1 and FGF/Xbra signal crosstalk in the germ layer specification and its inhibitory role for early neurogenesis in mesoderm and ectoderm regions. The implications of such a crosstalk in *Xenopus* between these two pathways in the synergistic activation of *ventx1*.*1* and the negative regulation of early neurogenesis relative to germ layer specification are discussed below.

**Figure 5:**
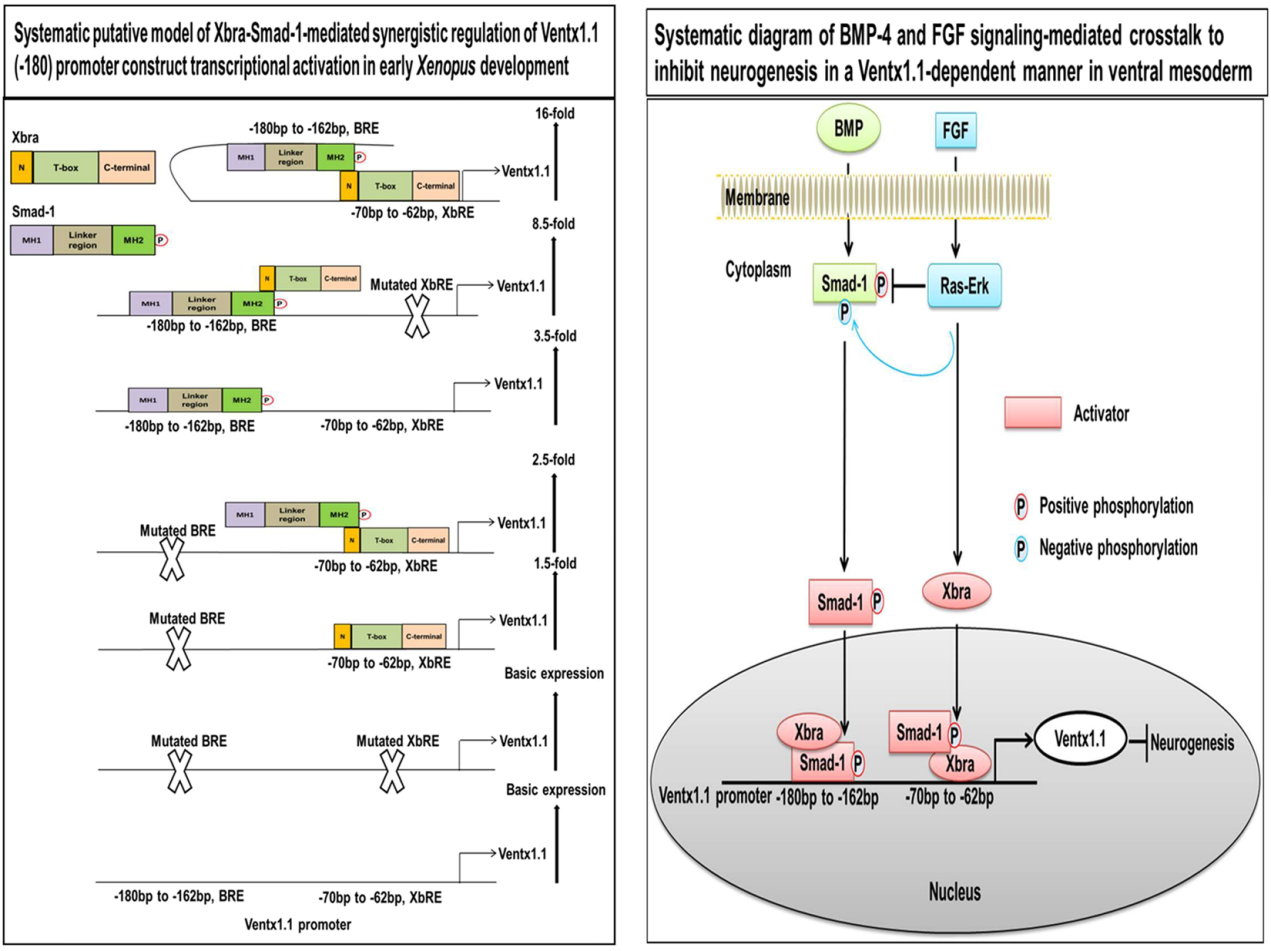
The putative model of Xbra-Smad-1-mediated synergistic regulation of *ventx1*.*1* transcriptional activation and neurogenesis inhibition in VMZ during early *Xenopus* development. The putative synergistic transcriptional activation of *ventx1*.*1* (−180) promoter is regulated by the physical interaction of Smad-1 and Xbra in *Xenopus* embryos. BMP-4/Smad-1 and FGF/Xbra mediate a crosstalk in VMZ, inhibiting neurogenesis in a Ventx1.1-dependent manner during embryonic development of *Xenopus*.

Our previous studies demonstrated that BMP-4/Smad-1 and FGF/Xbra separately induce *ventx1*.*1* expression in the ectoderm and mesoderm^4,14^. In the present study, we examined the mechanism of the synergistic activation of *ventx1*.*1* expression due to BMP-4/Smad-1 and FGF/Xbra pathways. Previously, a study had reported that Smad-1 physically interacts with Xbra in *Xenopus*^19^. That study suggested that Smad-1-Xbra interaction may induce the expression of ventral genes. However, a direct target gene for Xbra-Smad-1 interaction was not reported. Here, we first tried to find an Xbra response cis-acting element in the *ventx1*.*1* promoter. Through serial deletion constructs of the *ventx1*.*1* promoter (Fig. 2c), we found that the *ventx1*.*1* (−103) promoter construct contained a putative cis-acting XbRE (ATCACACTT, within −70 bp~−62 bp). The site-directed mutagenesis confirmed that the putative XbRE positively regulates *ventx1*.*1* transcription activation in *Xenopus* embryos (Fig. 2f,g), and the ChIP-PCR assay confirmed that Xbra directly binds and stimulates the transcription of *ventx1*.*1* (Fig. 2i,j). Although, we observe that XbRE-mutated *ventx1*.*1* (−103) mXbRE promoter construct contains at least one negative cis-acting response element(s) where a putative transcription factor increased by Xbra may bind. However, we do not know what it can be at this moment. In the beginning, we suspected that ventx1.1 itself may be the auto-negative regulatory factor (based on ChIP-Seq data and reporter gene assays with *ventx1*.*1* (−2525). However, *ventx1*.*1* negative response was abolished with *ventx1*.*1* (−399 and smaller) constructs. As such, we do not know the identity of the factor. Overexpression of Xbra and Smad-1 synergistically cooperated to activate *ventx1*.*1* transcription in the *ventx1*.*1* (−103) construct which did not contain the BRE (Fig. 3a,b,f). The site-directed mutagenesis showed that an intact BRE without XbRE also plays a significant role in Smad-1 and Xbra-mediated synergistic activation of *ventx1*.*1* transcription (Fig. 3e). We also documented the contribution of each cis-acting acting element (BRE and XbRE) to the *ventx1*.*1* promoter activity by studying signal changes in presence and also absence of the other interacting trans-acting element (Smad-1 and Xbra) (Fig. 5). Xbra and Smad-1 interaction enhanced the binding of an interacting partner on its cis-acting element (Fig. 4) and maximum cooperation was achieved in presence of intact DNA binding sequences for both Smad-1 and Xbra (Fig. 3j). It was also noted that Smad-1 and Xbra did not synergistically cooperate to activate transcription of another direct target of BMP/Smad-1, namely Xvent2, in *Xenopus* embryos (Fig. S1a and S1b). The present study collectively concludes that both the protein interaction between Xbra and the C-terminal phosphorylated Smad-1 and the DNA binding enhancement of Xbra and Smad1 within the *ventx1*.*1* promoter establish a signaling crosstalk between the BMP-4/Smad-1 and FGF/Xbra pathways.

Our previous studies reported that Ventx1.1 was an endogenous neural inhibitor in *Xenopus* through the suppression of organizer genes and early neural genes including *chordin*, *noggin*, *foxD5a/b*, *gsc* and *zic3* via its C-terminal^5,7,8^. In this paper, we demonstrated that Xbra induced *ventx1*.*1* expression in a BMP-4-inhibited condition and suppressed the expression of early and late neural genes including *foxD5a*, *Ngnr*, *N-CAM* and *Otx2* in animal cap explants (Fig. 1). Studies have documented that inhibition of Xbra by DN-Xbra reduces *ventx1*.*1* expression and triggers the neurogenesis in animal cap explants of *Xenopus* embryos^14,26^. As Xbra is not expressed in the ectodermal animal pole region and does not play a role in the neural gene expression in ectoderm region, the DN-Xbra construct eliciting neural tissue formation in ectoderm was an enigma. Our present results demonstrate that Xbra directly upregulates *ventx1*.*1* expression which is involved in inhibition of neurogenesis in animal cap explants; this suggests that ectopic expression of DN-Xbra may be involved in inhibition of *ventx1*.*1* expression, leading to neural tissue formation in ectoderm.

Presence and absence of Xbra determines whether FGF/MAPK is inhibitory or activating in the target tissue. In mesoderm, FGF/MAPK induces Xbra which activates *ventx1*.*1* expression, resulting in inhibition of neurogenesis^14,15^. FGF/MAPK enhances neural induction by inhibiting BMP-4/Smad-1 signaling in the ectoderm^15^. However, FGF-mediated BMP-4/Smad-1 inhibition alone is not sufficient to trigger neurogenesis in *Xenopus* embryos in animal cap explants^3^. In our previous and present study, FGF/Xbra induced *ventx1*.*1* expression in animal cap explants, resulting in neural inhibition and the knockdown of *ventx1*.*1* induced neurogenesis in FGF-treated ectodermal explants^14^. Xbra triggers and maintains the mesoderm and it seems that Xbra plays a role in regulating *ventx1*.*1* expression in the mesoderm and that if *ventx1*.*1* is upregulated, neural tissue formation is inhibited in the mesoderm region. Our study suggests that FGF/Xbra negatively regulate neurogenesis in a Ventx1.1-dependent manner in the mesoderm region. Xbra induces *ventx1*.*1* expression and inhibits the expression of early neural genes, leading to neural inhibition in part of mesodermal region (ventral and lateral mesodermal zone) of *Xenopus* embryos. Our study suggests that in *Xenopus* embryos, venxt1.1 plays neural inhibitory roles both in the ectoderm as a target of BMP and in part of the mesoderm as a target gene of FGF with gradient of BMP signaling.

The first main event in embryogenesis is germ layer specification where we hypothesize that the immediate early transcription factors expressed by the gradients of main signaling pathways including TGF-β (Vg1, nodal and activin), BMP and FGF maintain the resultant germ layer and suppress the commitment genes of other germ layers. In the mesoderm, Xbra is a main transcription factor elicited by BMP, FGF and nodals/activin via both direct and indirect signaling cascades. In the present paper, we suggest that BMP-4/Smad-1-mediated Ventx1.1 inhibits neurogenesis in the ectoderm and BMP-4/Smad-1 and FGF/Xbra mediated Ventx1.1 inhibits neural tissue formation in ventral and lateral mesoderm of *Xenopus* embryos. We also found that Xbra induces *ventx1*.*1* expression in a BMP-4-inhibited condition in animal cap explants of *Xenopus* embryos (Fig. 1a). Xbra-induced *ventx1*.*1* expression may play an essential role in inhibition of neurogenesis via suppressing the expression of early neural genes, including *FoxD5a* and *Zic3* in animal cap explants (Fig. 1b). As we expected, knockdown of *ventx1*.*1* restored the Ventx1.1-inhibited expression of neural genes, including *FoxD5a*, *Otx2*, *Ngnr* and *N-CAM* (Fig. 1c,d). These studies strongly suggest that Xbra stimulates *ventx1*.*1* expression and inhibits neurogenesis in a Ventx1.1-dependent manner in the ventral and lateral mesoderm regions.

In this paper, we also hypothesize that the immediate early transcription factors expressed in a specific germ layer suppress the commitment genes of other germ layers. We suggest that Ventx1.1 in the ectoderm is involved in repression of neuroectoderm genes (*FoxD5a/b* and *Zic3*) and organizer genes (*noggin*, *chordin*, and *gsc*). Due to the involvement of Ventx1.1 in neural inhibition, we performed a genome-wide ChIP-Seq of Ventx1.1 in *Xenopus* and found that Ventx1.1 directly binds to the promoter regions of neural and organizer specific genes, including *Zic3*, *noggin*, *chordin*, *FoxD5b* and *gsc*, repressing their mRNA expression (unpublished data). Xbra may thus participate in *ventx1*.*1* expression in the ventral and lateral mesoderm and maintain the mesoderm character. On the other hand, in the neuroectoderm, BMP inhibition leads to expression of *foxd5* and *zic3* as early neural genes. *Ventx1*.*1* (−103) reporter construct contains a negative response element for FoxD5b (Fig. 2f). In genome-wide ChIP-Seq of FoxD5b, FoxD5b directly binds within the promoter regions of ectoderm and mesoderm specific genes including *ventx1*.*1* (unpublished data). It will be interesting to study that the reciprocal repression networks existing between germ layer specific repressor factors. We consider *ventx1*.*1* to be an appealing candidate to investigate as a reciprocal repressor in the ectoderm versus FoxD5b in the neuroectoderm or for *ventx1*.*1* in the ventral and lateral mesoderm versus Gsc in the organizer region. During germ layer specification, a newly transcribed repressor in a specific germ layer preoccupies the region and inhibits the other germ layer specific genes that include essential genes for commitment and maintenance of the germ layers.

Germ layer specification is dependent on various cross talking pathways. In this regard, the FGF pathway is one of these main signaling routes concerning the regulation of germ layer specific genes. Our previous study demonstrated that Activin/Smad-2 and FGF signaling regulate the conversion of dorsal-ventral mesoderm formation in *Xenopus* embryos^27^. In presence of FGF signaling, Activin/Smad-2 stimulates DMZ formation and inhibits *ventx1*.*1* and α-globin expression. Meanwhile, in the absence of FGF signaling, Activin/Smad-2 converts the DMZ into VMZ and induces the expression of *ventx1*.*1* and α-globin in animal cap explants^27^. It will be interesting to investigate *ventx1*.*1* expression and its interaction with various signal cross talks in a context dependent fashion. In this paper, we demonstrated the cooperation of BMP/Smad-1 and FGF/Xbra to positively regulate *ventx1*.*1* expression. Smad-1 is activated by its C-terminal phosphorylation when BMP binds to hetero-dimeric BMP receptors. On the other hand, activation of FGF/Ras/MAPK pathway leads to phosphorylation of the Smad-1 linker region, known to inactivate Smad-1. In this paper, we demonstrated that C-terminal phosphorylation of Smad-1 is required for protein interaction with Xbra protein (Fig. 3i) and that the C-terminal phosphorylated mimic of Smad-1 (Smad-1(3SD)) showed a stronger affinity to Xbra than that of wild type Smad-1 (Fig. 3i, lane 1 and 2). Smad-1 affinity changes to Xbra, a co-Smad (Smad-4) or other transcription factors and whether Smad-1 changes affinity for its cis-acting element(s) in absence or presence of FGF signaling needs to be studied. These could answer how BMP-4 signaling with/without FGF differently regulates the expression of germ layer specific genes including *ventx1*.*1* in *Xenopus* embryos. The mechanism uncovered in this paper is similar to that reported by Trompouki *et al*. (2011) and Mullen *et al*. (2011), in which a master tissue regulator or another transcription factor directs Smad-1 to specific targets depending on the tissue. We found that a mesoderm specific transcription factor Xbra directs stronger binding of Smad-1 onto *ventx1*.*1* promoter in Xbra overexpressed animal cap explants (a mesoderm tissue). Since *ventx1*.*1* transcription is tightly regulated in different germ layers, it will be interesting to investigate the specificity of Smad-1 binding to target genes in different germ layers and under different sets of signaling combinations^28,29^. In addition, it will be interesting to examine the effects of a master neuro-ectoderm transcription factor, namely FoxD5b, and the dorsal mesoderm or organizer specific transcription factor, Gsc, on interaction and binding of Smad-1 to *ventx1*.*1* promoter.

Recently, a study documented that BMP-4 induces the expression of hematopoietic genes, including *ventx1*.*1*, *SCL* (Stem cell leukemia), *Xvent1/2*, *globin* and *GATA1/2/3*, leading to hematopoiesis in human and *Xenopus* embryos^30^. Other studies have demonstrated that elevated levels of GATA2 and Xvent2 are strongly associated with myeloid cell differentiation and acute myeloid leukemia (AML) progression in human cells^31,32^. Knockdown of *GATA2* and *Ventx* inhibits the AML cell growth and proliferation *in vivo* and *in vitro*. In the present study, we found that Ventx synteny and putative Smad-1 and Xbra binding response elements were evolutionarily conserved for the Xvent family members in human and *Xenopus* cells (Fig. S2a and S2b). The above studies suggested that the interactions of Ventx family genes with master transcription factors (R-Smads, TCF7l1/2, and C/EBPα) may regulate the fate of embryonic stem cells (ESCs) and hematopoietic progenitor cells (HPCs) and direct the differentiation of cells into specific lineages in vertebrates. Taken together, we propose a putative model of Xbra-Smad-1-mediated synergistic regulation and transcriptional activation of *ventx1*.*1* in the ventral and lateral mesoderm, inhibiting the neurogenesis in a Ventx1.1-dependent manner in early *Xenopus* embryogenesis (Fig. 5).

## Materials and Methods

### Ethics Statement

Institutional Animal Care and Use Committee (IACUC) approval is not required for the experimental use of amphibians or reptiles in Korea. All the members of our research group attended both the institutional educational and training courses for appropriate care and usage of experimental animals. Adult *X*. *laevis* were grown in 12-hour light/dark (LD 12:12 h) cycles at 18 °C according to the guidelines of Institutes of Laboratory Animal Resources for laboratory animal maintenance. Methods of DNA and RNA preparation, embryo injection, animal cap assay, luciferase assay, immunoprecipitation, site-directed mutagenesis, RT-PCR, and ChIP-PCR assay are available as supplementary information accompanying the manuscript.

## Electronic supplementary material

Supplementary Information
