# Supplementary material for: Xbra and Smad-1 cooperate to activate the transcription of neural repressor ventx1.1 in Xenopus embryos
Source: Sci Rep. 2018 Jul 30;8:11391. doi: 10.1038/s41598-018-29740-9 (PMC6065435; doi:10.1038/s41598-018-29740-9)
Supplement: Supplementary file 1 — Supplementary Information [file 41598_2018_29740_MOESM1_ESM.pdf]

Xbra and Smad-1 cooperate to activate the transcription of neural repressor *ventx1.1* in *Xenopus* embryos

Shiv Kumar[1], *Zobia Umair*[1], *Jaeho Yoon*[1], *Unjoo Lee*[2], *SungChan Kim*[1], *Jae-Bong Park*[1], *Jae-Yong Lee*[1], *Jaebong Kim*[1*]

[1]*Department of Biochemistry, Institute of Cell Differentiation and Aging, College of Medicine, Hallym University, Chuncheon, Gangwon-Do, 24252, Republic of Korea*

[2]*Department of Electrical Engineering, Hallym University, Chuncheon, Gangwon-Do, 24252, Republic of Korea*

***Address correspondence to:** Department of Biochemistry, Institute of Cell Differentiation and Aging, College of Medicine, Hallym University, Chuncheon, Gangwon-Do, 24252, Republic of Korea. Fax: +82-33-244-8425; Tel: +82-33-248-2544; E-mail: jbkim@hallym.ac.kr

Physical interaction of Smad-1 and Xbra uniquely regulates *ventx1.1* transcription, but not *Xvent2* (*Xom*), in the Xvent family.

Previous studies have suggested that both Xbra and Smad-1 interaction may induce the expression of ventral genes, leading to embryo ventralization [1,2]. Moreover, Xbra and Smad-1 separately induce *Xvent2* expression in animal cap explants of *Xenopus* embryos [3,4]. We thus examined whether the Xbra-Smad-1 interaction may also synergistically cooperate to regulate the transcriptional activation of *Xvent2*. We co-injected the *Xvent2* (-1031) promoter construct with Xbra and Smad-1, in combination or separately. Unlike *ventx1.1*, the Smad-1 and Xbra interaction did not synergistically cooperate to increase the relative promoter activity of *Xvent2* (Fig. S1a). In addition, Xbra decreased Smad-1-induced relative promoter activity of the *Xvent2* (-1031) promoter compared to *Xvent2* (-1031)-Smad-1 co-injected embryos (Fig. S1a, bar 3-4). This reduction might be from Xbra-mediated activation of FGF/MAPK signaling in a positive feedback loop. FGF/MAPK signaling is known to support neurogenesis by inhibiting Smad-1 activity [5-7]. Furthermore, FGF can also induce the expression of early neural genes, including *Zic3* and *FoxD5b,* which trigger neurogenesis in *Xenopus* embryos [8-10]. Shown with RT-PCR, the Xbra and Smad-1 interaction did not synergistically induce *Xvent2* expression (Fig. S1b). However, both Xbra and Smad-1 induced *Xvent2* expression in *Xenopus* embryos, separately. These results collectively indicate that the *ventx1.1* may be one of the unique targets of Xbra-Smad-1 interaction among the Xvent family transcriptional factors.

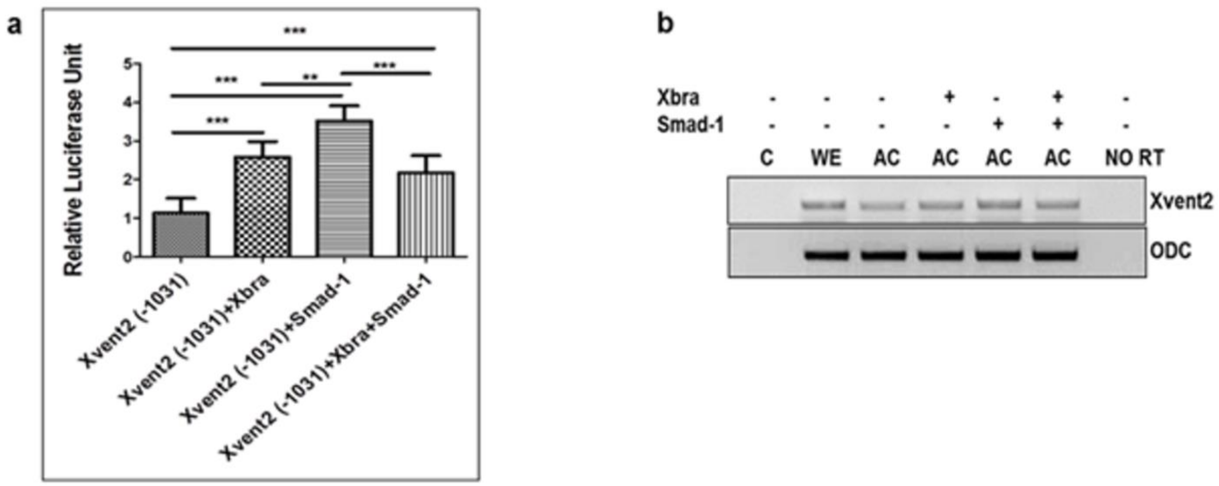

Supplementary Figure 1. Physical interaction of Smad-1 and Xbra uniquely regulates *ventx1.1* transcription activation, but not that of *Xvent2*, in the Xvent family. We co-injected the *Xvent2* promoter region construct with Xbra and Smad-1, in combination or separately, at the 1-cell stage and dissected the animal cap of injected embryos at stage 8. Dissected animal caps were also harvested in L-15 culture media until stage 11, followed by RT-PCR and reporter gene assay of the samples. (**a**) *Xvent2* (-1031) promoter region injected with Xbra and Smad-1, in combination or separately. (**b**) Embryos were co-injected with Xbra and Smad-1, separately or in combination to perform the RT-PCR at the stage 11. All relative promoter activity experiments were performed in triplicate. All relative promoter activity data are shown as mean ± SE.

Supplementary figure 2a

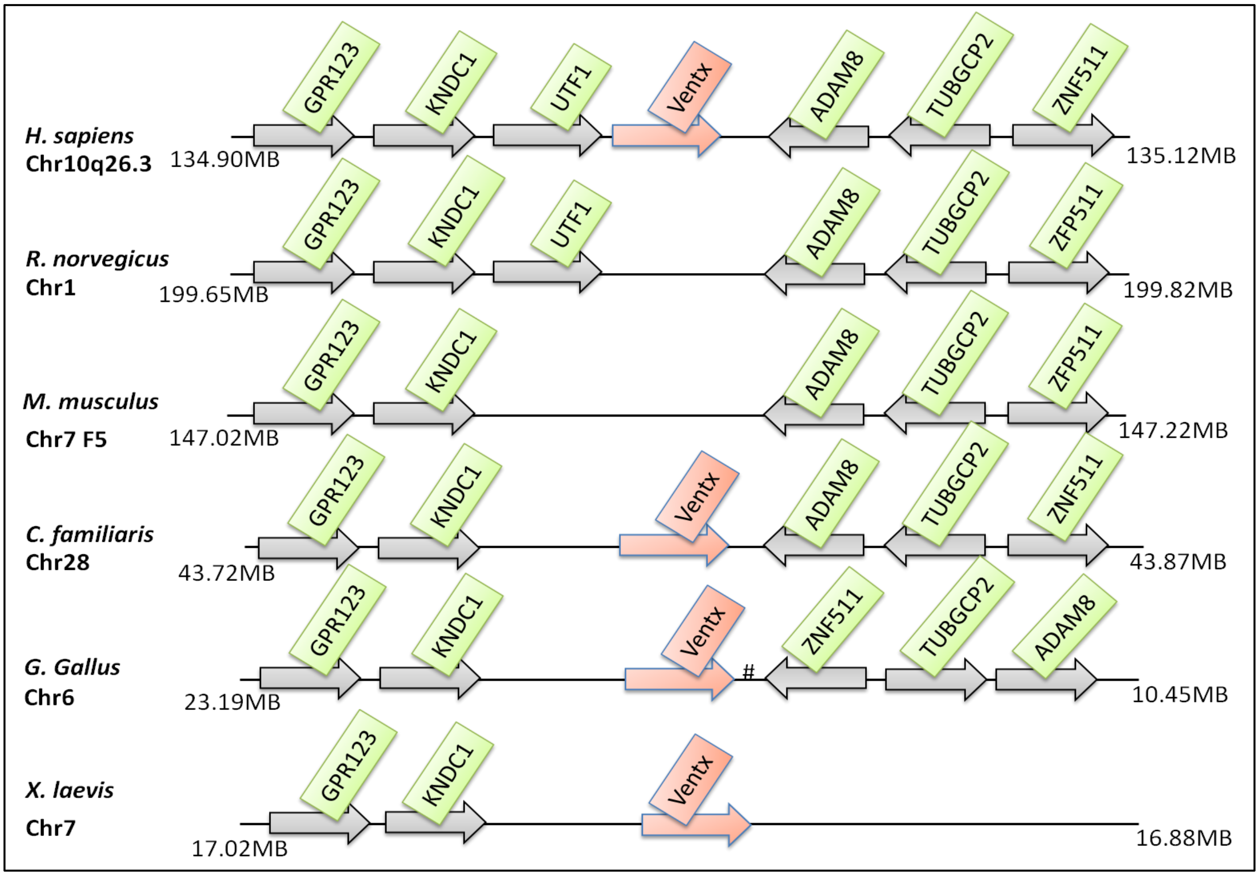

Supplementary Figure 2a. Chromosomal localization of *ventx* gene synteny in human, dog, chicken, and *Xenopus* genomes. Ventx synteny is evolutionarily conserved in human (*H. sapiens*), dog (*C. familiaris*), chicken (*G. Gallus*), and frog (*X. laevis*) genomes while Ventx synteny has been lost in rat (*R. norvegicus*) and mouse (*M. musculus)* genomes.

Supplementary figure 2b

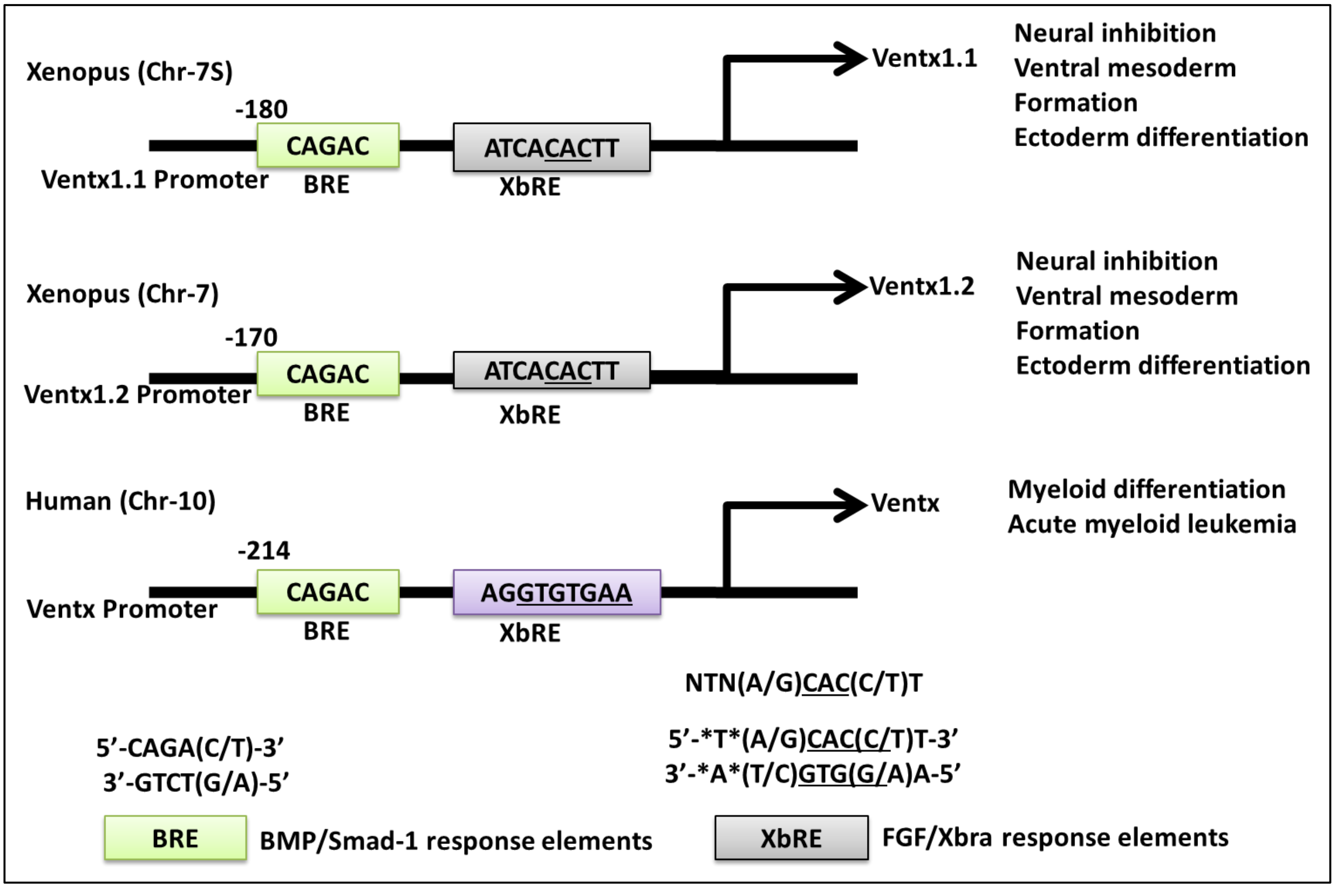

Supplementary Figure 2b. Putative Smad-1 (BRE) and Xbra (XbRE) binding response elements might be evolutionarily conserved in the promoter region of *ventx1.1s* (*Xenopus*) and *VENTX* (human). Putative BRE and XbRE may be evolutionarily conserved within the proximal promoter region of *ventx1.1s* and *ventx1.2* (*Xvent1*) in *Xenopus* as well as in *VENTX* for human. Violet color indicates complementary binding site for Xbra within the promoter of *Ventx*.

Fig. 1a

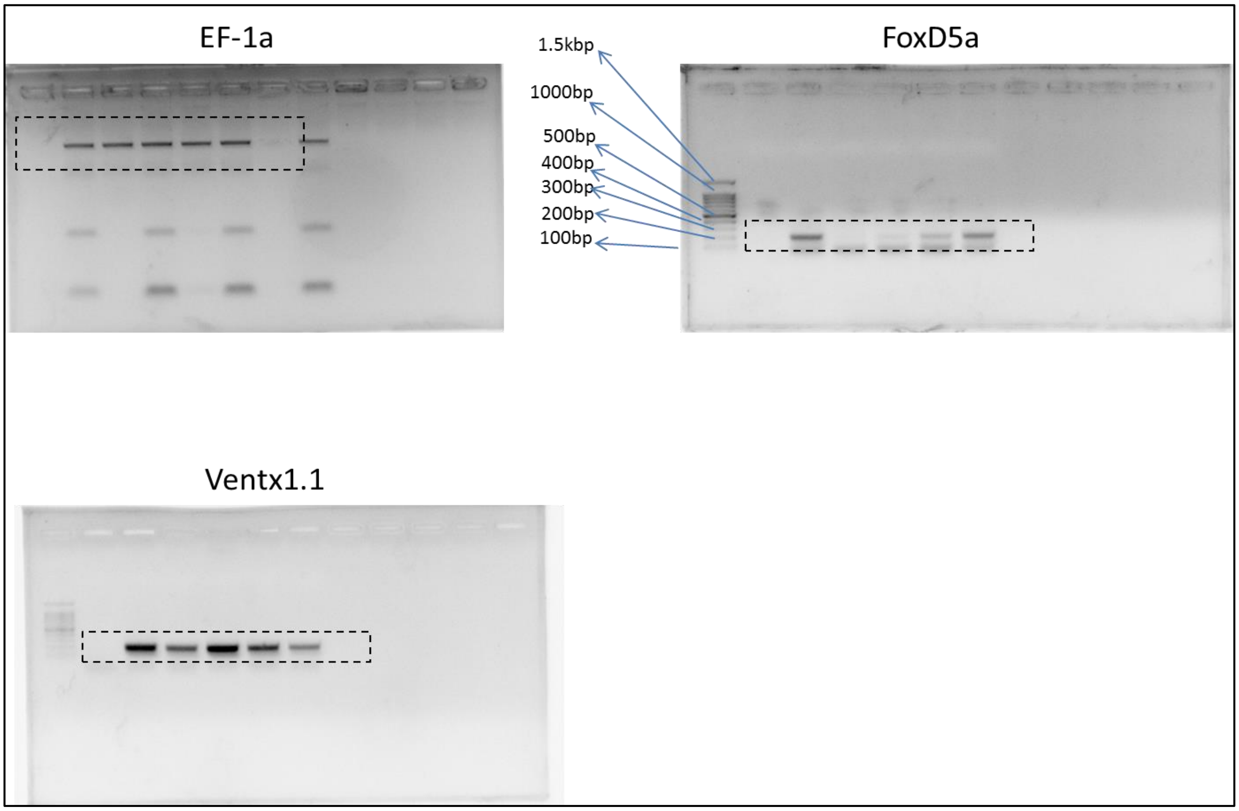
EF-1a
1.5kbp
1000bp
500bp
400bp
300bp
200bp
100bp
FoxD5a
Ventx1.1

Fig. 1b

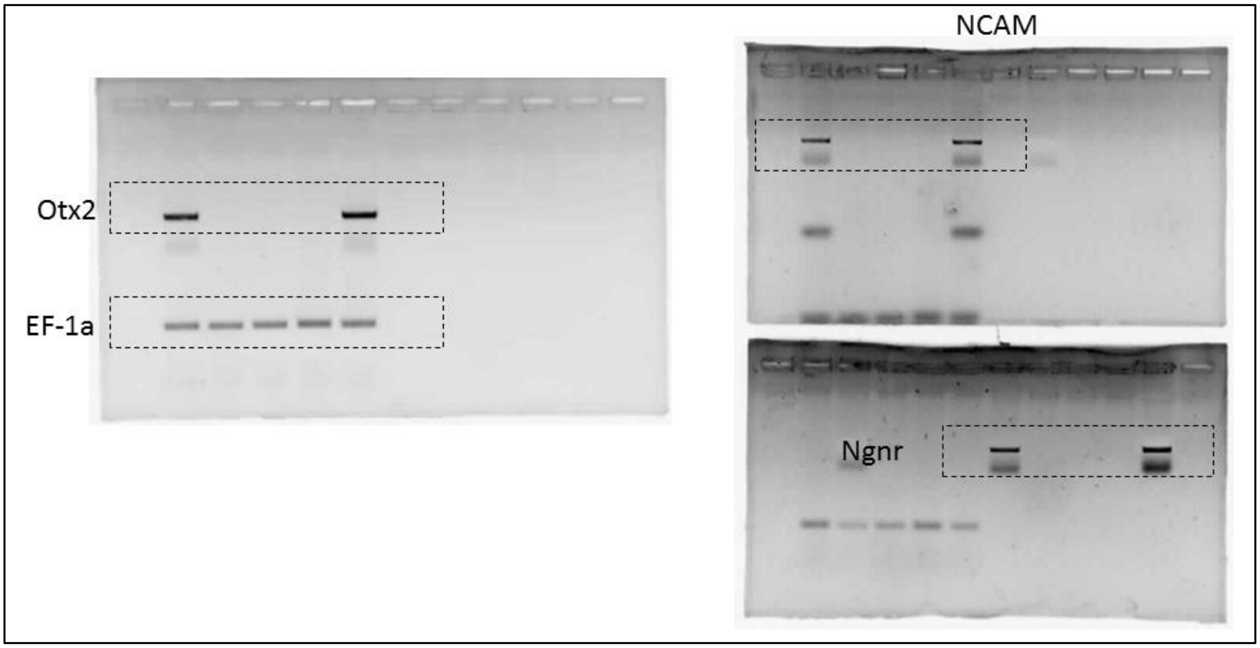
NCAM
Otx2
EF-1a
Ngnr

Fig. 1c

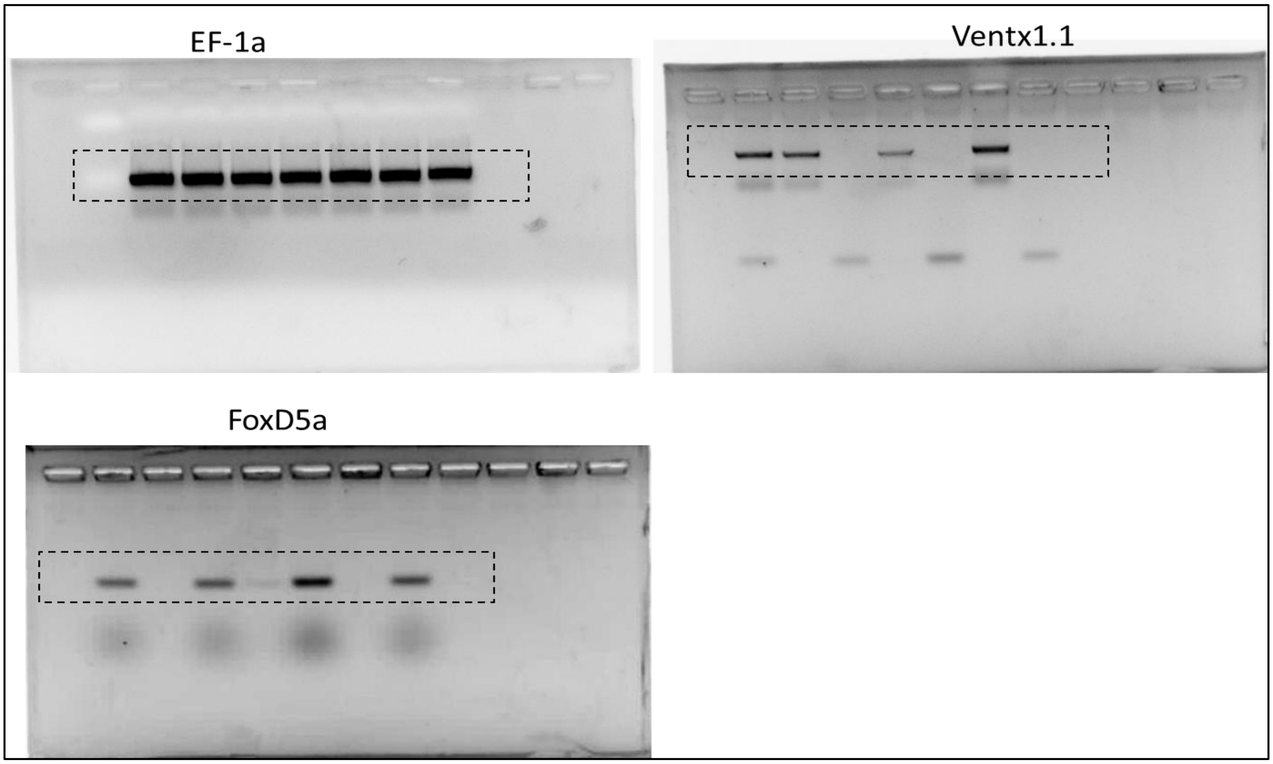
EF-1a
Ventx1.1
FoxD5a

Fig. 1d

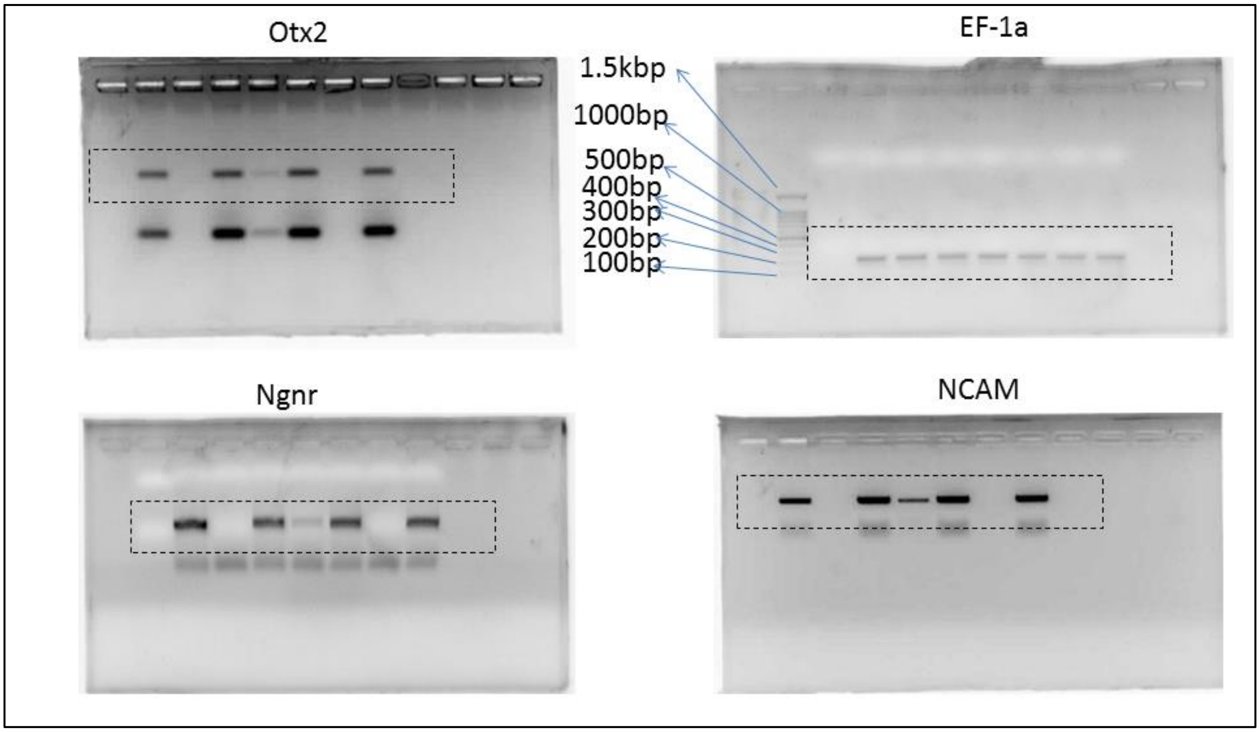
Otx2
EF-1a
1.5kbp
1000bp
500bp
400bp
300bp
200bp
100bp
Ngnr
NCAM

Figure S3. Full length gel pictures in Figure 1 (a-d).

Dashed boxes indicate the portion of gels included in the figures.

Fig. 2i

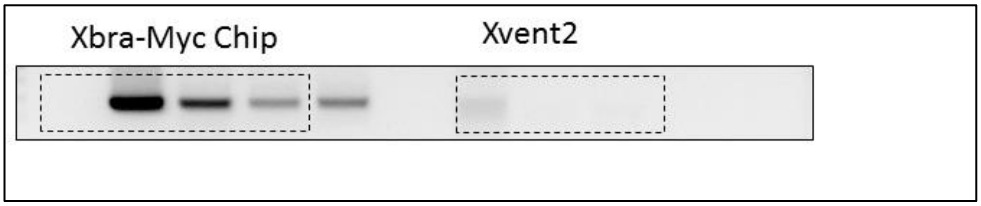

Figure S4. Gel picture of Xbra-Myc ChIP-PCR and negative control *Xvent2* in Figure 2i.

Dashed boxes indicate the portion of gel pictures included in the figures.

Supplementary Fig. 1b.

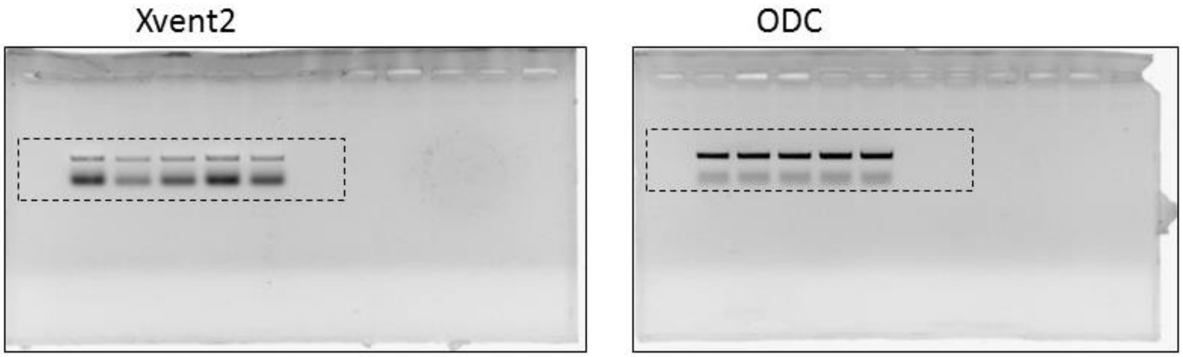

Figure S5. Full-length gel pictures in supplementary Figure 1b.

Dashed boxes indicate the portion of gel pictures included in figures.

Fig.4 a.

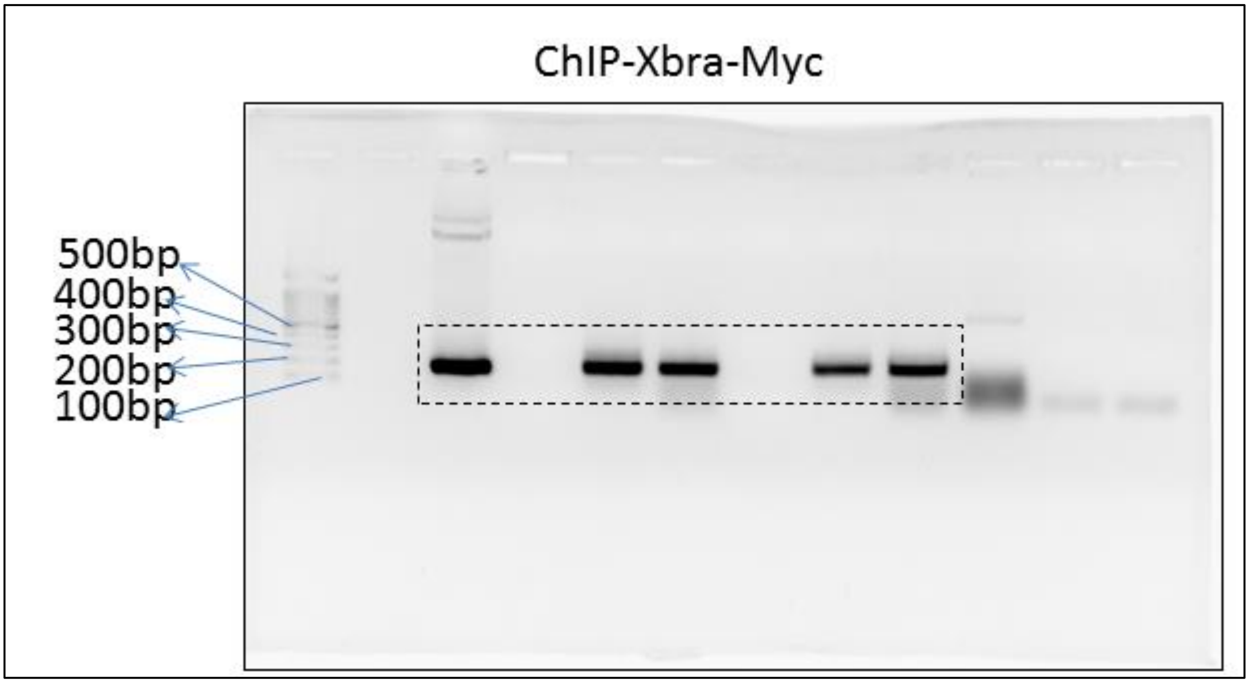

Fig. 4b

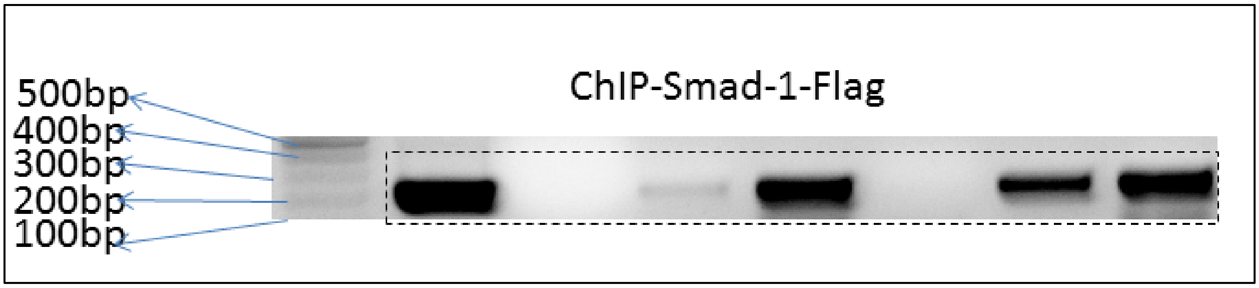

Fig. S6. Full-length gel pictures for Figure 4a and Figure 4b).

Dashed boxes indicate the portion of gel pictures included in the figures.

Materials and Methods

Ethics statement

Institutional Animal Care and Use Committee (IACUC) approval is not required for the experimental use of amphibians or reptiles in Korea. All members of our research group attended

both the institutional educational and training courses for appropriate care and usage of experimental animals. Adult *X. laevis* were grown in 12-hour light/dark (LD 12:12 h) cycles at 18^0C according to the guidelines of the Institutes of Laboratory Animal Resources that are tasked for laboratory animal maintenance.

DNA and RNA preparation

cDNAs encoding dominant-negative BMP receptor (DNBR), Flag-Smad-1 (WT): SP6, Asp718, and its mutants Flag-Smad-1 (3SD): SP6, Asp718, and Flag-Smad-1 (3SA): SP6, Asp718, HA-FoxD5b: SP6, Asp718 were all subcloned into the pSP64T expression vector while Myc-Xbra: SP6, Asp718 was subcloned into the pCS2+ expression vector [11]. Each vector was linearized with the appropriate restriction enzyme and used for in-vitro transcription using the MEGAscript kit according to manufacturer's instructions (Ambion, Austin, TX). Synthetic mRNAs were quantified by spectrophotometer at 260/280nm (SPECTRA max, Molecular Devices).

Cloning of *ventx1.1s* (*Xvent-1b, PV.1*) genomic DNA

The cloning of *ventx1.1* genomic DNA (gDNA) of Chr-3S was performed into the pBluescriptSK(-) plasmid (Stratagene, Cedar Creek, TX) as described by Lee et al., 2011 [11].

***ventx1.1* promoter constructs**

The 2.5 kb of 5'-flanking region of positive clone was subcloned into the pGL-2 basic plasmid (Promega, Madison, WI) and was designated as the -2525 bp construct. Serially-deleted *ventx1.1* promoter constructs and triple-repeat BMP-4-response element (BRE) were generated from the -2525 bp construct and subcloned into a pGL-2 basic plasmid by PCR amplification (Table 1) and according to Lee et al. 2011 [11].

Embryo injection and explants culture

Xenopus laevis were obtained from the Korean *Xenopus* Resource Center for Research. *Xenopus* embryos were injected after in vitro fertilization of eggs that was induced by injection of 500 units of human chorionic gonadotropin (Sigma, St. Louis, MO). DNAs (40 pg/embryo) and RNAs (1 ng/embryo) were injected into the animal pole at 1-cell stage embryos and harvested into 30% MMR. Then, animal cap (AC) dissected from injected embryos at stage 8.0-8.5 and incubated in 1X L-15 growth medium (Gibco/Thermo Fisher, Waltham, MA) until stage 11 and 24 for RT-PCR.

RNA isolation and RT-PCR

The mRNA (1 ng) was injected into the animal pole at 1-cell stage of *Xenopus* embryos and harvested into the 30% MMR solution with respect to control non-injected embryos until stage 8. Animal caps were then dissected from the injected and non-injected embryos and incubated until stage 11 or 24 into the 1X L-15 growth medium. Total RNA was isolated from whole embryos or animal caps using RNA-bee reagent following the manufacturer's instructions (TEL-TEST, Friendwood, TX) and treated with DNase I for removal of gDNA contamination. RT-PCR was performed with Superscript II (Invitrogen, Carlsbad, CA), as described by the manufacturer, with 2 mg total RNA per reaction. PCR was performed according to the following conditions: 30 seconds at 94^{0}C, 30 seconds at each annealing temperature, 30 seconds at 72^{0}C; 20-30 cycles of amplification (Table 2).

Luciferase assays

Relative luciferase reporter gene activities were measured using a luciferase assay system according to manufacturer's instructions (Promega, Madison, WI). Five different groups of embryos (3 embryos per group) were harvested and homogenized in 10 µl lysis buffer per animal embryos. Ten (10) µL embryos homogenate were assayed with 40 µL luciferase substrate and

determined the reporter gene activity by a luminometer (EG & G Berthold, Bad Wildbad, Germany). All experiments were repeated at least three times using independently derived sample sets.

Immunoprecipitation

Embryos were co-injected with Myc-Xbra mRNA at the one-cell stage with Flag-Smad-1, Flag-Smad-1 (3SD), and Flag-Smad-1 (3SA) in three different groups and injected embryos were collected at stage 11. They were then homogenized in lysis IP buffer (1 M Tris [7.4]), 150 mM NaCl, 1% NP-40, 10% Triton-X, 0.5M EDTA, 50% glycerol, 50 mM NaF, 1 mM Na_3VO_4, 15 mM glycerophosphate and 200X phosphatase inhibitors (PMSF [Sigma, cat-7626], pepstatin A [Sigma, cat-P4265], leupeptin [Sigma, cat-L0649], and benzamidine [Sigma, cat-B6506]). Cell lysates were cleared by centrifugation and cleared lysate incubated with the c-Myc polyclonal antibody (Santa Cruz Biotechnology, Dallas, TX, SC-789) for overnight at 4^0C, immunocomplexes were precipitated by using protein A/G beads plus (Santa Cruz Biotechnology, SC-2003). The proper amount of precipitated beads-protein complex was boiled in sample buffer, and resolved by electrophoresis on 10% SDS-polyacrylamide gels. Western blotting of Flag-Smad-1, Flag-Smad-1(3SD), and Flag-Smad-1(3SA) was performed by using an anti-Flag monoclonal antibody (Sigma, F-1804) and a secondary antibody anti-mouse (Stressgen, San Diego, CA, SAB-100). Immune complexes were visualized by using an ECL detection kit (GE Healthcare, Little Chalfont, United Kingdom).

Chromatin immune-precipitation (ChIP)

Chromatin immunoprecipitation assay was performed as described [12]. Embryos were injected at the one-cell stage with mRNA encoding Myc-Xbra and Flag-Smad-1 (1 ng per embryo) either separately or in combination. Injected embryos were collected at the stage 11 (100

embryos/sample) and processed according to the protocol. Polyclonal C-Myc (Santa Cruz Biotechnology, SC-789) and anti-Flag monoclonal antibody (Sigma, F-1804) were added to immunoprecipitate the chromatin. Moreover, normal rabbit IgG (Santa Cruz Biotechnology, SC-2027) and normal mouse IgG (Santa Cruz Biotechnology, SC-2025) were used as controls. PCR were performed with immunoprecipitated fragmented chromatin using *ventx*1.1 (-180 and -103) promoter region primers. Primers are shown in Table 1 and 2.

Site-directed mutagenesis

Mutagenesis was performed by a site-directed mutagenesis (Muta-Direct™) (iNtRON Biotechnology, Seongnam, Korea) kit using several oligonucleotides in accordance with instructions (Table 3).

Morpholino Oligoes (MOs)

Ventx1.1 morpholino oligoes (MOs, Genetools, LLC) were anti-sense oligo deoxynucleotides used for the loss-of-function study. This morpholino was designed against the 5' UTR and/or the start site of transcription initiation of the gene [4]. The sequence of *ventx1.1* MOs is as follows:

ventx1.1 MOs: 5'-GTCAATAGAGAATCCCTGTTGAACC-3'

MOs were warmed at 55^0C for 5 min and keep at 37^0C until injection of the embryos to avoid the clogging of microinjection needles. *ventx1.1* MOs were injected with 10 ng per embryos.

Nucleotide sequence accession number

v*entx1.1* (accession number; AF133122) cDNA sequence has been submitted to GenBank.

Statistical analysis

These data were analyzed by GraphPad Prism4 program. Statistical analysis involved one-way ANOVA. A $p < 0.05$ was considered to be significant. **: $p \leq 0.01$, ***: $p \leq 0.001$, n.s.: not significant.

Tables

Table 1. Primers used for serially-deleted reporter gene constructs

	Primer name	Sequences (5' ⟶ 3')
Upstream primers	-2525	AGTCCTCGAGTACCTGCAACTTACTCGC
	-300	AGTCCTCGAGAACCTACATTATCTCTTTCC
	-262	AGTCCTCGAGTCTCTGCTGTCTGTCCATGGGA
	-240	AGTCCTCGAGTTCTGTGCCGGCCAATGCTAAT
	-204	AGTCCTCGAGCCTCCAATATCACAAGGTGAA
	-180	AGTCCTCGAGACTAACCTGACAGACTCACTGG
	-103	AGTCCTCGAGTAGCCCATTCTGATAGCC
Downstream primer		AGTCAAGCTTGATGGAGCCGCTGGAGTTGTG

Table 2. Primers used for RT-PCR amplification

Gene name	Sequence (5' ⟶ 3')	Annealing temp (^{0}C)	Cycles
N-CAM	F-5'-CACAGTTCCACCAAATGC-3'<br>R-5'-GGAATCAAGCGGTACAGA-3'	57	29
FoxD5a	F-5'GACAGTGAGATGCTGAGTCC-3'<br>R-5'GGACTCTGCAGGATAGCCT-3'	50	30
Ngnr	F-5'GGATGGTGCTGCTACCGTGCGAGTACC-3'<br>R-5'CAAGCGCAGAGTTCAGGTTGTGCATGC-3'	65	30
Ventx1.1	F-5'CCTTCAGCATGGTTCAACAG-3'<br>R-5'CATCCTTCTTCCTTGGCATCTCCT-3'	60	27
Xvent2	F-5'CTACAGCACTAGCACTGACTCAGG-3'<br>R-5'TTGGACTGCATGCTGCAATACAGG-3'	57	25
Otx2	F-5'GGATGGATTTGTTGCACCAGTC-3'<br>R-5'CACTCTCCCAGCTCACTTCTC-3'	57	27
EF-1α	F-5'CAGATTGGTGCTGGATATGC-3'<br>R-5' ACTGCCTTGATGACTCCTAG-3'	57	20

Table 3. Primers used for site-direct mutagenesis gene constructs

Mutated site	Name	Primer Name	Sequences

XbRE	XbRE(M)	mXbRE-F	5’-CCCTTTGATGTGGATCAC*G*CTTGAATATCCATCAAGC-3’
		mXbRE-R	3’-GGGAAACTACACCTAGTG*C*GAACTTATAGGTAGTTCG-5’
BRE	-180 MT	mBRE-F	5’-AGTCCTCGAGACTAACCTGAC*CA*ACTCACTGG-3’
		mBRE-R	3’-TCAGGAGCTCTGATTGGACTG*GT*TGAGTGACC-5’

References

1 Marcellini, S. When Brachyury meets Smad1: the evolution of bilateral symmetry during gastrulation. *BioEssays : news and reviews in molecular, cellular and developmental biology* **28**, 413-420, doi:10.1002/bies.20387 (2006).

2 Messenger, N. J. *et al.* Functional specificity of the Xenopus T-domain protein Brachyury is conferred by its ability to interact with Smad1. *Developmental cell* **8**, 599-610, doi:10.1016/j.devcel.2005.03.001 (2005).

3 Lee, H. S. *et al.* Transcriptional regulation of Xbr-1a/Xvent-2 homeobox gene: analysis of its promoter region. *Biochemical and biophysical research communications* **298**, 815-823 (2002).

4 Yoon, J. *et al.* PV.1 induced by FGF-Xbra functions as a repressor of neurogenesis in Xenopus embryos. *BMB reports* (2014).

5 Alarcon, C. *et al.* Nuclear CDKs drive Smad transcriptional activation and turnover in BMP and TGF-beta pathways. *Cell* **139**, 757-769, doi:10.1016/j.cell.2009.09.035 (2009).

6 Bluemn, E. G. *et al.* Androgen Receptor Pathway-Independent Prostate Cancer Is Sustained through FGF Signaling. *Cancer Cell* **32**, 474-489 e476, doi:S1535-6108(17)30407-5 [pii]10.1016/j.ccell.2017.09.003 (2017).

7 Sapkota, G., Alarcon, C., Spagnoli, F. M., Brivanlou, A. H. & Massague, J. Balancing BMP signaling through integrated inputs into the Smad1 linker. *Molecular cell* **25**, 441-454, doi:10.1016/j.molcel.2007.01.006 (2007).

8 Janesick, A. *et al.* ERF and ETV3L are retinoic acid-inducible repressors required for primary neurogenesis. *Development* **140**, 3095-3106, doi:dev.093716 [pii]10.1242/dev.093716 (2013).

9 Rogers, C. D., Moody, S. A. & Casey, E. S. Neural induction and factors that stabilize a neural fate. *Birth defects research. Part C, Embryo today : reviews* **87**, 249-262, doi:10.1002/bdrc.20157 (2009).

10 Wilson, S. I., Graziano, E., Harland, R., Jessell, T. M. & Edlund, T. An early requirement for FGF signalling in the acquisition of neural cell fate in the chick embryo. *Current biology : CB* **10**, 421-429 (2000).

11 Lee, H. S. *et al.* Direct response elements of BMP within the PV.1A promoter are essential for its transcriptional regulation during early Xenopus development. *PloS one* **6**, e22621, doi:10.1371/journal.pone.0022621 (2011).

12 Blythe, S. A., Reid, C. D., Kessler, D. S. & Klein, P. S. Chromatin immunoprecipitation in early Xenopus laevis embryos. *Developmental dynamics : an official publication of the American Association of Anatomists* **238**, 1422-1432, doi:10.1002/dvdy.21931 (2009).
